# Effect of Osteoblast-Derived Extracellular Vesicles on Osteosarcoma Cells’ Transcriptional Profile: Role of Shuttled miRNAs

**DOI:** 10.3390/biomedicines14051039

**Published:** 2026-05-03

**Authors:** Luca Giacchi, Argia Ucci, Veronica Zelli, Chiara Compagnoni, Elisa Pucci, Alessandra Tessitore, Marco Ponzetti, Nadia Rucci

**Affiliations:** Department of Biotechnological and Applied Clinical Sciences, University of L’Aquila, 67100 L’Aquila, Italy; luca.giacchi@graduate.univaq.it (L.G.); veronica.zelli@univaq.it (V.Z.); chiara.compagnoni@univaq.it (C.C.); elisa.pucci@graduate.univaq.it (E.P.); alessandra.tessitore@univaq.it (A.T.); mp.univaq@gmail.com (M.P.); nadia.rucci@univaq.it (N.R.)

**Keywords:** osteosarcoma, osteoblast, extracellular vesicles, microRNA/miRNA, transcriptomics/transcriptome

## Abstract

**Background/Objectives**: Osteosarcoma is the most common primary malignant bone tumour, affecting children and young adults. Recent evidence suggests that extracellular vesicles (EVs), small membrane-bound nanoparticles released by all cell types, play a key role in intercellular communication within the tumour microenvironment. Therefore, we aimed to investigate the effects of osteoblast-derived EVs (OB-EVs) on osteosarcoma cell behaviour and to characterise the transcriptional and miRNA-mediated mechanisms underlying these effects. **Methods**: Phenotypic assays were performed to assess metabolic activity, proliferation, apoptosis, and invasion ability of human osteosarcoma cell lines after treatment with OB-EVs. Illumina-based RNAseq was conducted on RNA isolated from OB-EVs-treated cells, and qRT-PCR was assessed using commercially available TaqMan miRNA cards on RNA isolated from OB-EVs. **Results**: In U2OS cells, OB-EVs reduced metabolic activity (1.30-fold decrease, *p* = 0.0137) and proliferation (1.70-fold decrease, *p* = 0.017) while increasing apoptosis (1.15-fold increase, *p* = 0.014). In MG63, OB-EVs increased proliferation (4.9-fold increase, *p* = 0.020) without affecting tumour cell aggressiveness, while normal osteoblast behaviour was not affected by OB-EVs. MNNG/HOS cells treated with OB-EVs for 48 h showed substantial transcriptomic changes, with 296 differentially expressed genes (97 up- and 199 down-regulated in OB-EVs treated cells versus untreated cells), indicating a direct impact of OB-EVs on gene expression. Intriguingly, Gene Set Enrichment Analysis (GSEA) showed trends consistent with modulation of signalling pathways, including Wnt/β-catenin and NOTCH. Conversely, miRNA profiling of OB-EVs identified 13 highly expressed miRNA. Integration of transcriptomic and miRNA target prediction data highlighted convergent pathway-level signals, suggesting that OB-EVs may modulate tumour-associated regulatory networks. **Conclusions**: Taken together, these findings indicate that OB-EVs modulate osteosarcoma cell phenotype, with miRNA shuttling representing a potentially relevant contributing mechanism. The integrative analysis suggests that pathways associated with proliferation and cellular homeostasis, including Wnt/β-catenin signalling, may be involved, although further functional validation is required to confirm these mechanisms.

## 1. Introduction

Among primary bone tumours, osteosarcoma is the most prevalent (20–40%), affecting mainly children and adolescents, with a median age of 15 years old at diagnosis [1] and a worldwide incidence of 2–3 million/year [2]. Osteosarcoma is a high-grade tumour that localises mainly in bone segments with the most extensive longitudinal growth, such as proximal tibia, distal femur and proximal humerus [1]. Osteosarcoma is typically managed in a multidisciplinary fashion, which includes surgical excision, radiotherapy and multiagent chemotherapy, underscoring the need to find better therapeutic options for patients [3]. The most dramatic consequences of this tumour are related to the high rate of recurrence and the poor prognosis of metastatic disease, with lungs being the most commonly affected sites [4]. Osteosarcoma typically arises from multipotent mesenchymal stem cells (MSCs) that have undergone malignant transformation, likely because of a defective osteogenic differentiation programme [5], eventually leading to the production of defective bone with osteoid deposition.

It is well known that tumour cells interact with the host tissue to promote their local growth and dissemination, but the cross-communication of osteosarcoma cells with the bone microenvironment is still an issue deserving to be more investigated. Bone is a complex microenvironment where osteosarcoma cells could liaise with different resident cells, including osteoclasts, osteoblasts, osteocytes, stromal cells and endothelial cells [6]. Understanding the molecular mechanisms that mediate the interaction between malignant cells and host tissue could be the key to identifying more efficacious therapeutic targets. To this point, we previously demonstrated a mutual crosstalk between osteosarcoma cells and osteoblasts by means of extracellular vesicles (EVs). A growing body of evidence demonstrated that EVs are biological nano-scale messengers released by nearly all cell types, which are recognised as an effective communication tool for tumour cells, being involved in tumour development, growth and invasion [7]. Extracellular vesicles shuttle a wide range of biologically active macromolecules, such as proteins, DNA, mRNAs and non-coding RNA, including miRNAs, lncRNAs, circRNAs and tRNAs [8]. Indeed, we found that human MNNG/HOS osteosarcoma-derived EVs are able to reprogramme osteoblast homeostasis, by reducing the expression of cell cycle and pro-osteoblastogenic genes, whilst increasing transcriptional expression and protein release of pro-osteoclastogenic/inflammatory cytokines [9]. Conversely, underscoring the potential bi-directionality of the crosstalk between osteosarcoma and the microenvironment through EVs, we also demonstrated the ability of osteoblast-derived EVs (OB-EVs) to influence MNNG/HOS cells’ phenotype. Specifically, we found OB-EVs to decrease MNNG/HOS metabolic activity, viability, motility and in vitro invasion, all very important mechanisms in malignancy [10].

In this work, we confirmed the antitumoural effect of OB-EVs on U2OS and MNNG/HOS osteosarcoma cells, then we ventured to find whether some of the observed phenotypical changes could be explained by changes in gene expression profile and studied the role of miRNAs in the phenomenon. Across multiple analyses, we found a significant downregulation of *CDKN1A*, *NCOR2*, *RPL17*, *SNRNP70* and *UBALD2* genes in OB-EVs treated MNNG/HOS cells, to be further explored in future studies, and we explored whether miRNAs contained in OB-EVs may participate in the regulation of pathways affected by OB-EVs treatment, including proliferation-associated pathways and, potentially, Wnt/β-catenin signalling.

## 2. Materials and Methods

### 2.1. Materials

Dulbecco’s-modified minimum essential medium (DMEM), Dulbecco’s Phosphate-Buffered Saline (DPBS), penicillin, streptomycin and trypsin were from Euroclone (Milan, Italy). Foetal bovine serum (FBS) was from Thermo Fisher Scientific (Waltham, MA, USA). Sterile plasticware was purchased from Falcon Becton–Dickinson (Cowley, Oxford, UK) and Euroclone. Ultracentrifuge tubes were from Beckman Coulter (Brea, CA, USA). The TRIzol reagent, primers and reagents for qRT-PCR were from Invitrogen (Carlsbad, CA, USA). The SensiFAST SYBR no-ROX kit was from Meridian Bioscience (Cincinnati, OH, USA). The RNAeasy kit was from Qiagen (Heidelberg, Germany), while the Illumina-based RNAseq was performed by BMR Genomics (Padova, Italy). The SYTORNASelect Green Fluorescent Cell Stain, the TaqMan Advanced miRNA Human Serum/Plasma Cards and the RevertAid First Strand cDNA Synthesis kit were purchased from Thermo Fisher Scientific. All other reagents were from Sigma-Aldrich (St. Louis, MO, USA).

### 2.2. Animals

Procedures involving animals were designed and performed in conformity with national and international laws and policies (European Economic Community Council Directive 86/609, OJ L 358, 1, 12 December 1987; Italian Legislative Decree 4.03.2014 n.26, Gazzetta Ufficiale della Repubblica Italiana no. 61, 4 March 2014; National Institutes of Health guide for the Care and Use of Laboratory Animals, National Institutes of Health Publication no. 85-23, 1985) and the Animal Research: Reporting of in Vivo Experiments (ARRIVE) guidelines. Animal procedures received approval by the Italian Ministry of Health authority (Approval no. 173/2016-PR).

### 2.3. Cell Lines

The human osteosarcoma cell lines MNNG/HOS, MG63 and U2OS and the human foetal osteoblastic cell line hFOB1.19 were purchased by the European Collection of Authenticated Cell Cultures (ECACC, Salisbury, UK). Cells were cultured at 37 °C, 5% CO_2_ in DMEM plus 10% FBS, 100 IU/mL penicillin, 100 μg/mL streptomycin and 2 mM L-glutamine.

### 2.4. Osteoblast Primary Cultures

Calvariae from 7-day-old CD1 mice were collected and subjected to three sequential enzymatic digestions with 1 mg/mL Clostridium histolyticum type IV collagenase and 0.25% trypsin, at 37 °C for 15, 30 and 45 min, respectively, under agitation. The supernatant from the first digestion was discarded, while those arising from the subsequent digestions were centrifuged at 300× *g* for 7 min, and cell pellets were resuspended in DMEM supplemented with 10% FBS and cultured separately. At confluence, cells were detached, pooled together and plated according to the experimental needs.

### 2.5. Extracellular Vesicles Isolation

Extracellular vesicles (EVs) from mouse primary osteoblasts (OBs) conditioned medium (CM) were isolated according to Ucci et al. and Ponzetti et al. [9,10]. At 80% of confluence, OBs were washed in DPBS and starved in serum-free DMEM for 24 h. OB-CM was then subjected to two sequential centrifugations, the first at 300× *g*, 4 °C for 5 min to remove dead cells and the second at 5000× *g*, 4 °C for 25 min to remove membrane debris. The latter supernatant was collected and ultracentrifuged at 100,000× *g*, at 9 °C for 70 min. The pellet, containing osteoblast-derived EVs (OB-EVs), was resuspended in DPBS. For quantification, OB-EVs were subjected to nanoparticle tracking analysis and protein extraction, the latter giving a yield of 4.9 ± 1.3 µg/12 mL CM.

### 2.6. MTT Assay

Metabolic activity of MG63 and U2OS cells was assessed by the 3-(4,5-dimethylthiazol-2-yl)-2,5-diphenyltetrazolium (MTT) bromide reduction assay. Both cell types were plated onto 96-well plates at a density of 9000 cells/cm^2^, starved overnight (O/N) in serum-free DMEM and then treated with OB-EVs isolated from one 175 cm^2^ flask (12 mL medium) or with DMEM (control). After 48 h of treatment, MTT dissolved in DPBS at 5 mg/mL concentration was added at a 1:6 (*v*/*v*) ratio directly into the cell supernatant. Three hours later, the medium was removed and DMSO was added to dissolve the precipitated formazan salts arising from the reaction. Plates were shaken at 160 rpm on an orbital shaker for 10 min, then absorbance at 595 nm was recorded.

### 2.7. EdU (5-Ethynyl-2′-deoxyuridine) Cell Proliferation Assay

MG63 and U2OS cells were plated onto 96-well plates at a density of 9000 cells/cm^2^ and, upon reaching 80% confluence, were starved O/N in serum-free DMEM and treated with OB-EVs isolated from one 175 cm^2^ flask (12 mL medium) or with DMEM (control). After 48 h, a cell proliferation assay was performed using the EdU HTS Kit 488 (Thermo Fisher Scientific, Waltham, MA, USA). Briefly, MG63 and U2OS cells were incubated for 5 h with 5 μM EdU (5-ethynyl-2′-deoxyuridine) in DMEM, washed in DPBS and fixed in 4% paraformaldehyde (PFA) for 10 min. Then, cells were permeabilised with 0.5% Triton X-100 in PBS, incubated with the click assay cocktail for 30 min at room temperature (RT) protected from light and then washed twice in 1× rinse solution. Nuclei were counterstained with DAPI (4′,6-Diamidino-2-phenylindole dihydrochloride). Cell proliferation was assessed under a fluorescence microscope and plotted as the percentage of proliferating cells (EdU-positive cells) over total cells.

### 2.8. Apoptosis

An Annexin Red assay was performed on MG63 and U2OS cells treated with OB-EVs by using the Guava^®^ Annexin Red Kit (#FCCH100108-Luminex, Austin, TX, USA). Cells were plated in cell culture dishes (100 mm Ø) at a density of 8000 cells/cm^2^, starved O/N in serum-free DMEM and then treated with OB-EVs (isolated from 12 mL of CM collected from one 175 cm^2^ flask) or with DMEM (control). After 48 h, conditioned medium was collected and cells were detached and centrifuged. The pellet was resuspended in a working solution with recombinant Annexin V conjugated to a red sensitive dye CF^®^647 for 15 min at 37 °C in an incubator. Samples were then analysed with the cytofluorimeter Luminex Easycyte (Luminex, Austin, TX, USA) equipped with a 642 nm laser.

### 2.9. Invasion Assay

An invasion assay was performed on MG63 and U2OS cells treated with OB-EVs. Briefly, cells were plated in cell culture dishes (100 mm Ø) and, upon reaching 80% confluence, were starved O/N in serum-free DMEM and treated with OB-EVs isolated from one 175 cm^2^ flask (12 mL medium) or with DMEM (control). After 48 h, both cell types were washed in DPBS, detached and centrifuged at 300× *g* for 5 min at RT. Each pellet was resuspended in serum-free DMEM and incubated for 30 min at 37 °C in 5% CO_2_. Then, 800,000 cells were seeded in the upper compartment of each transwell, onto an 8.0 µm membrane pre-coated with Matrigel, while 0.8 mL of FBS was added to the lower compartment as a chemoattractant. After 8 h for U2OS and 24 h for MG63, cells migrated towards the lower compartment of the transwells and were fixed with cold methanol, washed in DPBS and stained with haematoxylin/eosin. The number of invading cells per field was evaluated.

### 2.10. RNAseq Analysis of MNNG/HOS Cells

MNNG/HOS were cultured to 75% confluence in cell culture dishes (100 mm Ø) and starved O/N. Cells were then treated with OB-EVs isolated from one 175 cm^2^ flask (12 mL medium), while untreated cells were used as control (N = 3). After 48 h of treatment, RNA was isolated using the RNAeasy kit (Qiagen, Heidelberg, Germany) and checked for integrity by agarose gel and purity by nanodrop before being sent for further analyses and Illumina-based RNAseq to BMR Genomics (Padua, Italy). After QC and alignment vs the reference transcriptome (*Homo sapiens*), the raw counts were normalised to obtain transcripts per million (TPM). The analysis yielded 227,818 transcripts, and those with TPM < 10 in at least 2 over 3 samples in either one or both the experimental groups were excluded, leaving 11,125 transcripts for the bioinformatic analyses.

### 2.11. Real Time RT-PCR

RNA from MNNG/HOS, U2OS and MG63 cells treated with OB-EVs or with DMEM (control) for 48 h was extracted using the TRIzol reagent, reverse-transcribed (2 μg) into cDNA using the RevertAid First Strand cDNA Synthesis Kit (Thermo Fisher Scientific, Waltham, MA, USA) and subjected to transcriptional analysis by RT-PCR using a SYBR green-based master mix no-ROX. Data were analysed via dedicated software (Light Cycler 96 SW 1.1, Roche Diagnostics GmbH, Mannheim, Germany) using the ∆∆Ct method, and *Gapdh* was chosen as the housekeeping gene.

### 2.12. SYTORNA Staining of Osteoblast-Derived Extracellular Vesicles

Isolated OB-EVs were incubated for 20 min at 37 °C with the SYTORNASelect Green Fluorescent Cell Stain (Thermo Fisher Scientific, Waltham, MA, USA), then the EVs were washed in PBS, followed by ultracentrifugation at 100,000× *g*, at 4 °C for 70 min. Finally, the OB-EV pellets were resuspended in DMEM and used to treat MNNG/HOS cells previously plated in chamber slides at a density of 18,000 cells/cm^2^. After 1, 6 and 18 h of treatment, cells were fixed in 4% PFA and slides were mounted with a DAPI-containing medium before microscope qualitative analysis.

### 2.13. miRNA Profiling of Osteoblast-Derived Extracellular Vesicles

Total RNA was extracted from OB-EVs and control medium using the Plasma/Serum RNA Purification Mini Kit (Norgen Biotek, Thorold, ON, Canada) following the manufacturer’s protocol. MiRNAs analysis was performed by quantitative Real-Time PCR (qRT-PCR) using TaqMan Advanced miRNA Human Serum/Plasma Cards (Thermo Fisher Scientific, Waltham, MA, USA), which allow the simultaneous assessment of the expression levels of 188 unique miRNAs. In this study, we focussed on the 136 miRNAs that showed 100% sequence identity between human and mouse. The analysis was performed by comparing OB-EV preparations (N = 3) with CM to account for any background that may be arising from the medium. Samples were analysed on a ViiA7 instrument (Applied Biosystems, Carlsbad, CA, USA), and data were processed by QuantStudio Software v 1.3 and Expression Suite software v 1.3 (Applied Biosystems). Synthetic ath-miR159a was spiked-in during RNA extraction and used as exogenous control for normalisation of data. MicroRNAs’ expression levels were evaluated by the ΔΔCt method. Manual quality check on PCR amplification plots was also performed. MiRNAs showing the highest expression levels in OB-EVs compared to medium were considered for further analyses.

### 2.14. Bioinformatic Analyses

The differentially expressed gene (DEGs), clustergram and principal component analysis (PCA) on the RNAseq set were performed by BMR genomics using standard R packages. For gene-set enrichment analysis, the TPM values provided by BMR genomics were first subjected to ENST-gene name conversion, and transcripts with no gene ID were excluded. After calculating the average LogFC for all transcripts, data were analysed using the online gene set enrichment analysis (GSEA) tool Webgestalt (https://www.webgestalt.org/, accessed on 27 December 2025). The top 10 enriched/depleted terms with *p* < 0.05 were plotted based on normalised enrichment score (NES). Kaplan–Meier survival analyses were conducted using the R2 Genomics Analysis and Visualization Platform (https://hgserver1.amc.nl/cgi-bin/r2/main.cgi?option=kaplan_main, accessed on 29 March 2026) and using data from database GSE42352 (Mesenchymal osteosarcoma, N = 127 https://pubmed.ncbi.nlm.nih.gov/22454324/, accessed on 29 March 2026). The cutoff mode adopted was “scan” in all analyses and the minimum group size was set at N = 8. The median was used as the separation point between high- and low-risk groups.

For the miRNA target analysis, Mienturnet (http://userver.bio.uniroma1.it/apps/mienturnet/, accessed on 5 August 2022), TargetScan (https://www.targetscan.org/vert_80/, accessed on 5 August 2022), DIANA (https://dianalab.e-ce.uth.gr/html/mirpathv3/index.php?r=mirpath, accessed on 5 August 2022) and miRDB (https://mirdb.org/, accessed on 5 August 2022) were used to predict the miRNA targets, and the predicted targets were manually or automatically compared with the significantly downregulated targets in RNAseq to find any alignment between the two datasets. MiRNet (https://www.mirnet.ca/miRNet/home.xhtml, accessed on 5 August 2022) was used to predict the Reactome pathways most likely to be affected by the predicted targets of the miRNAs present in OB-EVs, which were then compared with the Reactome terms identified in the RNAseq. The overlapping pathways are the most likely to be affected by miRNAs in OB-EVs-treated MNNG/HOS and were therefore manually identified and presented.

### 2.15. Statistics

Numerical and statistical analyses were performed using GraphPad Prism version 9.0 (GraphPad Software, San Diego, CA, USA). Data are reported as mean ± SD. Statistical significance between two groups was assessed using one sample *t*-test or paired Student’s *t*-test, as specified in the figure legends. A *p*-value ≤ 0.05 was considered statistically significant.

## 3. Results

### 3.1. Osteoblast-Derived EVs Influence Osteosarcoma Cells’ Aggressiveness Without Affecting Normal Osteoblasts

**Figure 1:**
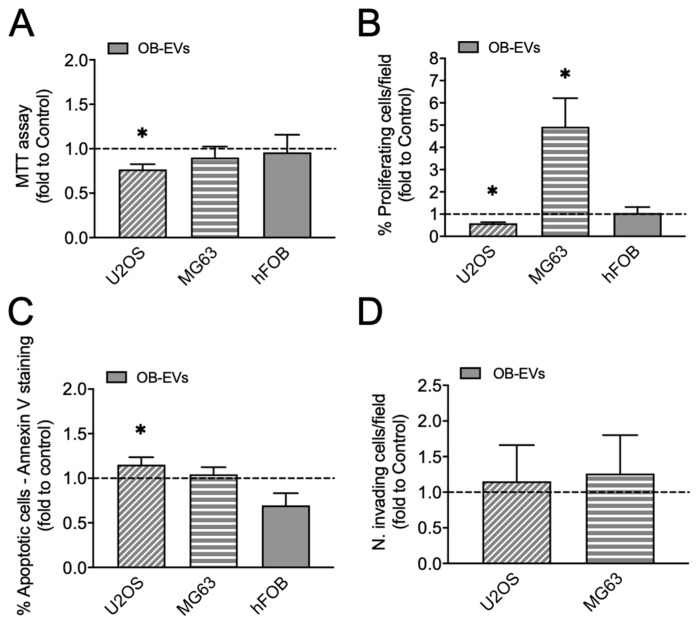
Effect of osteoblast-derived EVs on U2OS, MG63 osteosarcoma cells phenotype and on normal osteoblasts. (**A**–**C**) The human osteosarcoma cell lines U2OS and MG63 and the human foetal osteoblast-like cell line hFOB were untreated or treated for 48 h with EVs isolated from mouse primary osteoblasts (OB-EVs). Evaluation of (**A**) metabolic activity by MTT assay, (**B**) proliferation by EdU assay and (**C**) apoptosis by Annexin V staining. (**D**) U2OS and MG63 cells were pretreated with OB-EVs for 48 h, then they were detached, resuspended in DMEM without serum and seeded on the upper basket of a matrigel-coated transwell to perform an invasion assay over FBS. Data are presented as mean fold change compared to control ± SD of at least three independent experiments. * *p* < 0.05, paired *t*-test.

In our previous work, we proved that osteoblast-derived EVs reduced MNNG/HOS osteosarcoma cells’ aggressiveness and viability through redox-dependent signalling pathways [10]. Therefore, we investigated whether this effect was also true in two additional human osteosarcoma cell lines, i.e., U2OS and MG63. We first characterised OB-EVs for their size and concentration by nanoparticle tracking analysis (Figure S1A). Moreover, Western blot analysis showed that OB-EVs’ protein lysates expressed the typical EVs’ markers, such as CD81, CD63 and Annexin II (Anxa2) (Figure S1B), while TEM analysis on isolated particles demonstrated their membrane integrity and vesicular nature (Figure S1C). Next, U2OS cells were treated with OB-EVs for 48 h, finding a significant reduction in metabolic activity (Figure 1A) as well as in proliferation (Figure 1B) compared to untreated cells. At variance with our previous results obtained on MNNG/HOS [10] and reported in Table S1, treatment of U2OS cells with OB-EVs slightly but significantly increased apoptosis (Figure 1C), while invasion was not affected (Figure 1D). Looking at MG63 cells, OB-EVs failed to affect metabolic activity (Figure 1A), while it significantly increased proliferation (Figure 1B). Conversely, no changes in apoptosis (Figure 1C) and invasion (Figure 1D) were observed in MG63 cells treated with OB-EVs compared to control cells. Taken together, these results show that treatment of different osteosarcoma cell lines with OB-EVs acts in a different manner, impairing tumour cells aggressiveness in MNNG/HOS and U2OS cells while promoting proliferation in the less aggressive MG63 cell line. Therefore, we asked whether OB-EVs could also influence normal cell behaviour by treating the human foetal osteoblast cell line hFOB 1.19 (from now hFOB). We found that this was not the case, as neither metabolic activity nor proliferation or apoptosis was altered (Figure 1). These data, summarised in Table S1, suggest a selective regulatory effect of OB-EVs on osteosarcoma cells.

### 3.2. Osteoblast-Derived EVs Modify the Transcriptional Profile of MNNG/HOS Osteosarcoma Cells

**Figure 2:**
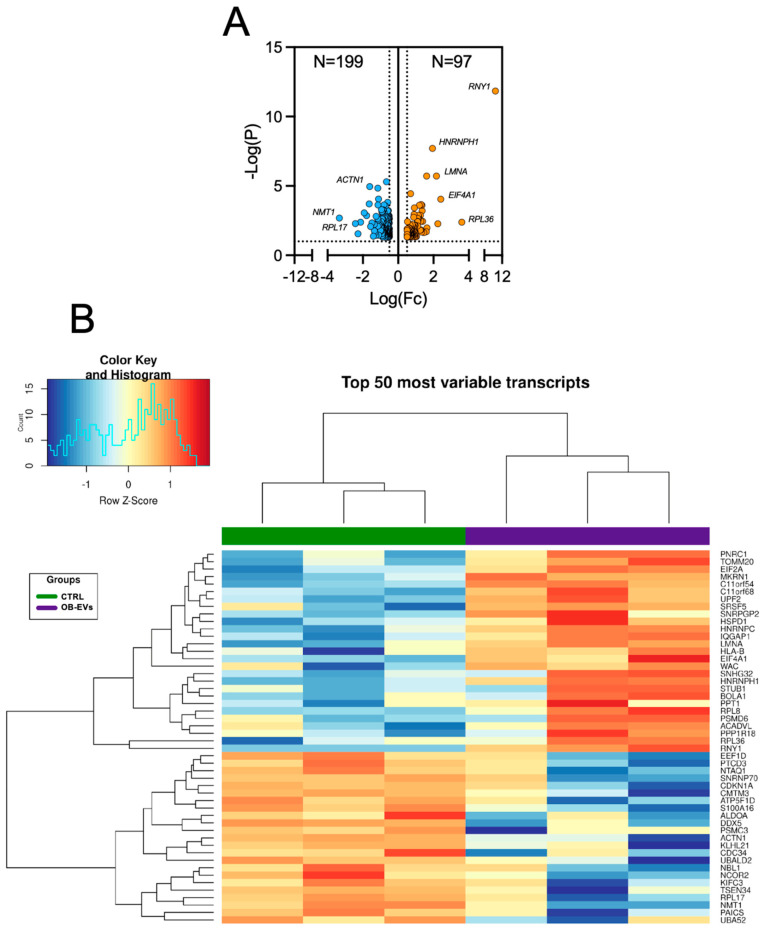
Differentially expressed genes (DEGs) in OB-EVs-treated versus untreated MNNG/HOS cells. (**A**) Volcano plot of differentially expressed genes (DEGs). The plot displays the distribution of DEGs between the two experimental conditions. The x-axis represents the Log fold change (LogFc), while the y-axis shows the −Log(*p*-value). Upregulated genes are highlighted in orange, downregulated genes in blue. The dotted lines represent the significance threshold. (**B**) Heatmap of the top 50 most variable transcripts. Hierarchical clustering of differentially expressed genes across samples, with expression levels represented as Z-score-normalised values (red: high expression; blue: low expression).

Based on this finding, we next asked whether OB-EVs could also influence osteosarcoma transcriptomic profile. MNNG/HOS cells, for which we have the deepest mechanistic insight on the effect of OB-EVs based on our previous work [10], were cultured in standard conditions or treated with OB-EVs for 48 h before extracting their RNA and performing RNAseq on them. Principal component analysis (PCA) of the transcriptomic profiles showed untreated and OB-EVs-treated MNNG/HOS cells clustered according to their treatment, indicating that the OB-EVs exerted a similar and detectable effect on all the biological replicates (Figure S2A); moreover, count distribution was similar among all samples (Figure S2B). In these three replicates-based experimental approaches, statistical analysis using *p* < 0.01 and a logFC > |0.5| as cut-off values revealed a total of 296 differentially regulated genes (DRGs) (Figure 2), of which 199 were downregulated (top 20 in Table 1) and 97 were upregulated (top 20 in Table 2). The complete list of significantly regulated genes is available in Table S2.

Focusing on RNA sequencing data, we performed gene set enrichment analyses (GSEA) on all transcripts that met quality criteria, without applying other cut-offs, as is commonly conducted. To this aim, we employed the WebGestalt tool (https://www.webgestalt.org/, accessed on 27 December 2025), to which we provided the gene IDs, previously converted from ENST IDs using gProfiler (https://biit.cs.ut.ee/gprofiler/convert, accessed on 5 August 2022), and the regulations as LogFC. KEGG GSEA (Figure S3A) revealed a significant enrichment of pathways related to fatty acid metabolism, protein processing in the endoplasmic reticulum and mTOR signalling, suggesting a strong modulation of cellular metabolic activity and protein homeostasis in OB-EVs-treated MNNG/HOS cells. Enriched pathways also included lipid and atherosclerosis-related processes and viral infection-associated pathways, which may reflect stress-related or inflammatory-like transcriptional programmes.

We then ran a similar analysis on Panther as a reference database (Figure S3B). The analysis highlighted a consistent depletion of pathways involved in integrin signalling, Wnt signalling, and inflammation mediated by chemokine and cytokine signalling. Additional negatively enriched pathways included CCKR signalling and Huntington disease-associated transcriptional programmes, suggesting broad alterations in signalling networks governing cell adhesion, migration and differentiation. No Panther pathways reached significant positive enrichment, reinforcing the predominance of pathway repression following OB-EVs treatment.

Intriguingly, Reactome GSEA (Figure S3C) further confirmed a strong enrichment of pathways associated with rRNA processing, RNA polymerase II-mediated transcription [11] and gene expression, including major nucleolar and cytosolic rRNA processing pathways, pointing to a marked remodelling of transcriptional and ribosomal biogenesis processes. Enrichment was also observed for cytokine signalling in the immune system, ISG15 antiviral mechanisms and energy metabolism integration, indicating activation of stress-response and immune-related transcriptional programmes. In contrast, a large set of pathways was significantly depleted, most notably those involved in cell cycle regulation, including G1/S transition, DNA damage checkpoints, and p53-dependent responses. Importantly, multiple NOTCH1 signalling pathways, including both canonical signalling and cancer-associated NOTCH1 mutant pathways, were consistently downregulated, highlighting a potential suppression of differentiation- and proliferation-related signalling axes.

Taken together, these data indicate that OB-EVs induce measurable but relatively modest transcriptional changes in osteosarcoma cells at this timepoint, which could be consistent with an early or intermediate regulatory response rather than a fully established downstream reprogramming.

**Figure 3:**
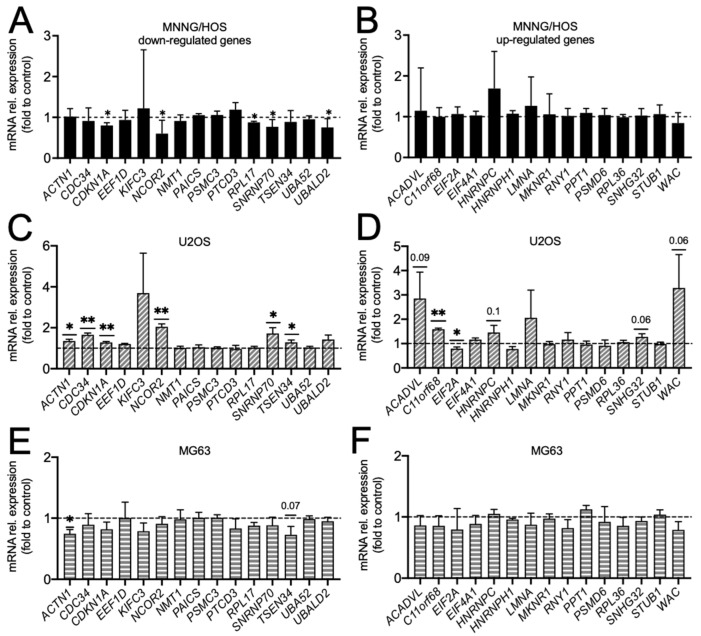
Effect of osteoblast-derived EVs on osteosarcoma cells’ transcriptional profile. The human osteosarcoma cell lines (**A**,**B**) MNNG/HOS, (**C**,**D**) U2OS and (**E**,**F**) MG63 were treated with EVs isolated from mouse primary osteoblasts (OB-EVs) or left in DMEM (control). RNA was extracted, reverse transcribed into cDNA and used for transcriptional analyses by RT-PCR (dot line = control set to 1) of the RNAseq-regulated genes (top 15 downregulated and top 15 upregulated in OB-EVs-treated MNNG/HOS vs untreated cells) indicated in the abscissa. Results are the mean ± SD of at least three independent experiments; * *p* < 0.05, ** *p* < 0.01, one sample *t*-test.

To validate our sequencing results, we evaluated by RT-PCR the expression of the top 15 downregulated (Figure 3A) and of the top 15 upregulated transcripts (Figure 3B) in OB-EVs-treated MNNG/HOS vs untreated cells. We confirm the downregulation of *CDKN1A* (*Cyclin dependent kinase inhibitor 1A*), *NCOR2* (*Nuclear receptor corepressor 2*), *RPL17* (*Ribosomal protein L17*), *SNRNP70* (*Small nuclear ribonucleoprotein U1 subunit 70*) and *UBALD2* (*UBA like domain containing 2*) genes (Figure 3A); however, no changes in the expression of the 15 most RNAseq upregulated transcripts were found (Figure 3B). We also analysed any modulation of the expression of these 30 genes in U2OS and MG63 treated with OB-EVs compared to untreated cells. Interestingly, and in contrast to what was achieved with MNNG/HOS cells, in U2OS treated with OB-EVs, we observed a significant increase in the expression of *ACTN1* (*Actinin alpha 1*), *CDC34* (*Cell division cycle 34*, *ubiquitin conjugating enzyme*), *CDKN1A*, *NCOR2*, *SNRNP70* and *TSEN34* (*tRNA splicing endonuclease subunit 34*) genes (Figure 3C), along with a reduced expression of the *EIF2A* (*Eukaryotic translation initiation factor 2A*) gene (Figure 3D). In agreement with the sequencing data, we observed an upregulation of *C11orf68* (*Chromosome 11 open reading frame 68*) gene, a trend of increase for *ACADVL* (acyl-Coenzyme A dehydrogenase, very long chain), *HNRNPC* (*Heterogeneous Nuclear Ribonucleoprotein C*), *SNHG32* (*Small Nucleolar RNA Host Gene 32*) and *WAC* (*WW domain-containing adaptor with coiled-coil*). Regarding the MG63 cell line, instead, we found a downregulation of *ACTN1* and a trend of decrease for *TSEN34* (Figure 3E), while all the other genes analysed were not modulated by OB-EVs treatment (Figure 3E,F).

Among the few downregulated genes whose modulation was also confirmed by real time RT-PCR, there was *RPL17*, which encodes for a structural component of the ribosomal subunit (large subunit), essential for protein synthesis. To assess its potential clinical relevance, we interrogated publicly available osteosarcoma patients’ databases, containing expression and survival data, through the R2 Genomics Analysis and Visualization Platform (https://hgserver1.amc.nl/cgi-bin/r2/main.cgi?open_page=login, accessed on 29 March 2026), discovering that low expression of *RPL17* correlates with higher overall survival (Figure S4A) and metastasis-free survival (Figure S4B). *RPL17* has never been previously associated with osteosarcoma, but these data are in line with the OB-EVs-mediated reduced aggressiveness previously observed in MNNG/HOS cells in vitro [10].

### 3.3. Osteoblast-Derived EVs Contain Micro-RNAs Predicted to Potentially Target MNNG/HOS Osteosarcoma Cells’ Transcripts

Current knowledge suggests that the most represented class of molecules in EVs is micro-RNAs (miRNAs), which have the potential to regulate multiple pathways at once, thus making them possible culprits for the biological regulations observed when treating cells with EVs [12,13,14,15]. Therefore, we performed a miRNA profiling in OB-EVs to find out which were the most highly represented miRNA species and to understand whether they could be mediating some of the effects observed in MNNG/HOS. We found that 27 miRNAs were expressed, which we define as miRNAs with a clean negative control, at least 4 Ct of difference between medium and OB-EVs, in all 3 OB-EVs preparations assayed (Data S1), while 13 of them were highly expressed in all OB-EVs preparations (Ct < 27.5, Table 3).

To assess whether such miRNAs were playing a role in molecular circuitries involving DRGs, we performed a target gene prediction analysis using the three different prediction web tools TargetScan release 8 (https://www.targetscan.org/vert_80/, accessed on 5 August 2022) accessed through Mienturnet (http://userver.bio.uniroma1.it/apps/mienturnet/, accessed on 5 August 2022), DIANA (https://dianalab.e-ce.uth.gr/html/mirpathv3/index.php?r=mirpath, accessed on 5 August 2022) and miRDB (https://mirdb.org/, accessed on 5 August 2022). Next, we checked whether any of the predicted targets matched the DRGs in OB-EVs-treated MNNG/HOS and then considered reliable predictions that were common to at least two of the three tested databases (Table 4).

**Figure 4:**
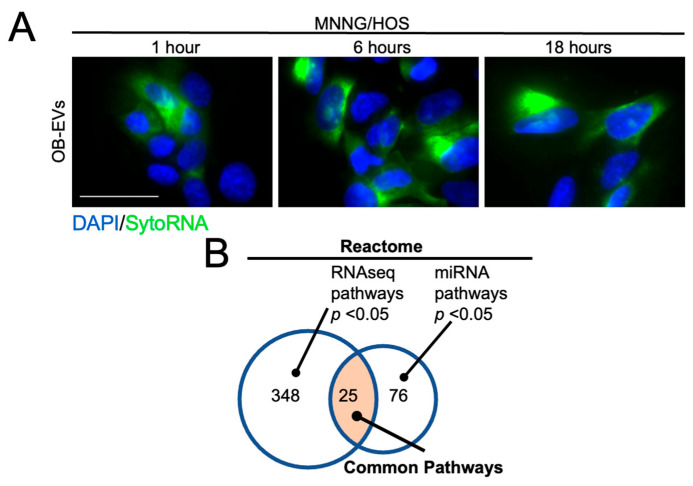
Potential effects of OB-EVs-expressed miRNAs on MNNG/HOS cells. (**A**) MNNG/HOS cells were treated for 1, 6 and 18 h with OB-EVs loaded with the RNA-specific green fluorescent dye, SytoRNA Select, and analysed, at the same timepoints, by epifluorescence microscopy. Cells’ nuclei were counterstained with DAPI. Scale bar = 50 µm. (**B**) Venn diagram illustrating the overlap between pathways experimentally regulated by OB-EVs (left circle) and those predicted to be influenced by OB-EV miRNAs (right circle), with 25 pathways meeting both criteria (orange). Each pathway was considered significant based on *p*-value  <  0.05.

Using this method, by primarily focussing the analysis on downregulated genes, we identified 37 potential target transcripts among the 199 downregulated, showing that 18% of them can have the potential of being also direct targets of the OB-EV-contained miRNAs. Interestingly, some of the target transcripts were predicted to be targeted by two (namely *ATL2*, *CDC34*, *CMTM3*, *COL1A1*, *EFHD2*, *ETV4*, *FN1*, *GALE*, *HDAC7*, *HMGA1*, *NCOR2*, *NPTN*, *PLXND1*, *PPP1R18*, *REEP4*, *RHOA*, *RRM2*, *SLC25A23*, *SMARCA4*) or three miRNAs (namely *SP1*, *TMEM135*, *ARL4C*). Knowing that miRNAs are among the most represented RNA species in EVs [16], we used an RNA-specific fluorescent dye (SYTO RNASelect) to tag RNAs contained in OB-EVs, and we then treated MNNG/HOS cells with the tagged EVs. Fluorescence microscopy analysis showed that OB-EVs RNAs were efficiently shuttled to MNNG/HOS cells (green-fluorescent dotted pattern) already 1 h after treatment, with a consistent signal achieved at 6 and 18 h from the treatment (Figure 4A).

As described before for the RNAseq, we decided to also have a more holistic look at the pathways regulated by OB-EVs, using miRNet (https://www.mirnet.ca/miRNet/home.xhtml, accessed on 5 August 2022) to understand the pathways that are most likely regulated by the miRNAs present in OB-EVs. We then reasoned that it would be important to understand the pathways that we found to be regulated by OB-EVs, and those that are predicted to be influenced by the miRNAs present in OB-EVs, and Reactome analyses carried out using this method showed that 25 pathways responded to these criteria (Figure 4B). From the integrative analysis, Wnt/β-catenin-related terms emerged among the most consistently represented pathways, with multiple Reactome entries identified in both the RNAseq and miRNet analyses (Table 5). Other interesting pathways regulated in both analyses were mitotic cell cycle and oncogene-induced cellular senescence.

Notably, although canonical core components of pathways such as Wnt/β-catenin or PI3K/AKT were not among the most strongly regulated transcripts, several differentially expressed genes can be functionally linked to these signalling axes. For example, *NCOR2* and *CDC34* are involved in transcriptional and ubiquitin-mediated regulatory mechanisms that may influence Wnt pathway activity, while *CDKN1A* and *ALDOA* are associated with cell cycle and metabolic processes downstream of PI3K/AKT signalling. In addition, the upregulation of translational regulators such as *EIF4A1* and *EIF2A* is consistent with modulation of mTOR-dependent pathways. Although these associations are indirect, they support the involvement of these signalling networks and are coherent with the pathway-level integration reported in Table 5.

Taken together, these results suggest that miRNAs contained in OB-EVs may contribute to the regulation of some of the pathways modulated by OB-EVs treatment, including Wnt/β-catenin-related signalling.

## 4. Discussion

Extracellular vesicles are well-known mediators of cellular communication, as they carry bioactive molecules that exert a direct effect on target cells, reprogramming their transcriptional and proteomic profile. Osteosarcoma is the most widespread primary bone tumour in childhood and adolescence, derived from a malignant transformation of MSCs and characterised by the ability to communicate with resident cells, which in turn increases its local growth and dissemination. In this study, we aimed to characterise the effect of osteoblast-derived extracellular vesicles (OB-EVs) on osteosarcoma cells, shedding light on the potential mechanisms and pathways influenced by their treatment. By integrating RNA sequencing, bioinformatics analyses and miRNA profiling, this work provides a comprehensive view of OB-EVs’ multiple roles, highlighting their potential as a tool for understanding osteosarcoma biology.

In our previous work, we observed that OB-EVs reduce the aggressiveness of MNNG/HOS osteosarcoma cells by impairing their viability, motility and invasion ability [10]. To assess whether these in vitro effects were reproducible across additional osteosarcoma models, we tested OB-EVs in U2OS and MG63 cells. It is important to underline that these frequently used cell models have been widely described in in vitro comparative studies for their different phenotypic features and molecular profile [17,18,19]. U2OS are generally described as adherent cells and often classified as fibroblastic because they are negative for many osteoblastic markers compared with more differentiated osteosarcoma models (i.e., Saos-2). By contrast, MG63 is one of the most heterogenous osteosarcoma cell lines, showing an oval to spindle-shaped morphology and an immature osteoblast-like or pre-osteoblastic phenotype, with a consistent variability in the expression of extracellular matrix-related markers that can translate into different degrees of aggressiveness in vitro. These cells can be more adherent, with more mesenchymal/osteogenic features compared to U2OS. These differences should be considered when interpreting functional assays, as the two lines can give different biological readouts and different or even opposite outcomes in response to the same treatment. In this regard, as previously observed in MNNG/HOS cells [10], OB-EVs significantly reduced metabolic activity of U2OS, indicating a consistent negative impact on cellular viability across distinct genetic backgrounds. However, U2OS cells exhibited a marked decrease in proliferation associated with a mild increase in apoptosis, consistent with a more pronounced growth-inhibitory response, while invasion was unaffected. With regards to MG63, OB-EVs treatment increased proliferation without affecting apoptosis nor invasive capacity. Importantly, when testing normal hFOB osteoblast-like cells, OB-EVs did not significantly alter metabolic activity, proliferation, or apoptosis. These data suggest that the anti-tumour effect of OB-EVs occurs primarily in highly aggressive cell lines (i.e., MNNG/HOS and U2OS), while it fades in less aggressive lines (MG63) or even has no impact on normal osteoblasts.

Having established that OB-EVs consistently modulate osteosarcoma cell phenotype, although with line-specific outcomes, we next investigated MNNG/HOS cells using RNA sequencing to define the transcriptional programmes underlying the response to OB-EVs. OB-EVs treatment induced a measurable transcriptomic reprogramming, identifying 296 differentially regulated genes, with a predominance of downregulated transcripts. To identify the most robust biological mechanisms driving this response, we prioritised a pathway-oriented integrative analysis (Table 5) rather than relying solely on enrichment approaches. By intersecting transcriptomic data with predicted targets of OB-EV-shuttled miRNAs, we highlighted convergent and biologically coherent signalling cascades. This approach allowed us to filter broader descriptive categories (including those derived from GSEA) and focus on pathways supported by independent lines of evidence. In this context, GSEA was interpreted as supportive evidence, as several enrichment trends were directionally informative but not consistently robust after multiple testing corrections. Notably, this integrative analysis revealed modulation of pathways related to cell-cycle regulation, apoptosis, and key oncogenic signalling axes, most prominently Wnt/β-catenin and PI3K/AKT, suggesting a coordinated impact on proliferation-associated transcriptional programmes, consistent with the observed phenotypic effects relevant to osteosarcoma progression.

Aberrant activation of the Wnt/β-catenin pathway has been extensively documented in osteosarcoma and is strongly associated with tumour initiation, progression, metastatic dissemination and resistance to therapy. Canonical Wnt signalling promotes osteosarcoma cell proliferation, survival, stemness and invasive behaviour by inducing the transcription of oncogenic targets such as *MYC*, *CCND1* and *AXIN2* [20,21]. Functional evidence further supports a pro-tumoural role for Wnt signalling in osteosarcoma, as genetic or pharmacological inhibition of β-catenin results in reduced proliferation, impaired migration and invasion and enhanced apoptosis of osteosarcoma cells both in vitro and in vivo [22,23]. In addition, Wnt signalling is critically involved in maintaining cancer stem cell-like properties in osteosarcoma, thereby contributing to tumour recurrence and chemoresistance [24]. In this context, the modulation of Wnt signalling highlighted by our integrative analysis provides a plausible pathway-level link between EV-associated molecular changes and the reduced metabolic activity and aggressiveness observed in MNNG/HOS cells.

In line with this, potential modulation of Wnt/β-catenin signalling may contribute to the reduced proliferation and aggressiveness observed in OB-EVs-treated MNNG/HOS cells.

Similarly, modulation of NOTCH-related signalling emerged from the broader analyses, although this observation was primarily derived from supportive approaches and should therefore be interpreted with caution. NOTCH signalling has a well-established pro-tumoural role in osteosarcoma, being associated with increased tumour growth, metastatic potential and poor clinical outcome [25,26,27,28]. In this context, the observed trends may be consistent with the overall reduction in proliferation-associated programmes but do not represent a central mechanistic conclusion of the present study.

Looking at the single genes, we examined the expression of the top 15 downregulated and 15 upregulated genes by RT-PCR in order to confirm our RNAseq dataset. We observed a partial validation of the dataset. This relatively modest transcriptional response window could particularly affect the robust detection of upregulated transcripts, as well as contribute to variability in downstream validation. Nonetheless, *CDKN1A*, *NCOR2*, *RPL17*, *SNRNP70* and *UBALD2* genes were successfully confirmed to be downregulated in OB-EVs-treated MNNG/HOS cells. This is consistent with the possibility that the RNA sequencing data capture an early or intermediate transcriptional response to 48 h OB-EVs exposure, which may not yet fully translate into stable downstream gene expression changes detectable by independent validation approaches.

Importantly, these gene-level changes were interpreted in the context of the integrative pathway analysis (Table 5). The validated genes form coherent functional clusters related to RNA processing, translation, anabolic activity, proteostasis and cell-cycle regulation, consistent with a reduced proliferative and biosynthetic state. For example, the downregulation of genes involved in protein synthesis and metabolism, such as *RPL17*, aligns with modulation of PI3K/AKT and mTOR-related processes. Although *RPL17* has not been previously investigated in the context of osteosarcoma, analysis of publicly available datasets suggests that lower *RPL17* expression is associated with improved clinical outcomes, supporting a potential link with reduced tumour aggressiveness. Similarly, *CDKN1A* (p21) is a key regulator of the cell cycle downstream of PI3K/AKT signalling. While its downregulation may appear context-dependent and requires further investigation, it likely reflects the complexity of regulatory networks affected by OB-EVs. *NCOR2*, a transcriptional co-repressor involved in chromatin regulation and known to interact with NOTCH signalling [29], further supports the modulation of transcriptional control mechanisms. *SNRNP70*, a core component of the U1 snRNP spliceosome with oncogenic functions in osteosarcoma [30], and *UBALD2*, implicated in ubiquitin-related processes, both point toward a broader impact on proteostasis and RNA processing pathways.

The profiling of miRNAs in OB-EVs identified 27 expressed miRNAs, 13 of which were highly expressed and putatively involved in mediating transcriptomic changes in OB-EVs-treated MNNG/HOS cells. Fluorescence microscopy confirmed rapid uptake of OB-EV RNA cargo by target cells, supporting the functional transfer of regulatory molecules. Integrative target prediction identified 37 out of 199 downregulated transcripts as potential direct targets of these miRNAs, suggesting that approximately 18% of transcriptional changes may be mediated by OB-EVs-derived miRNAs.

Notably, several predicted targets are involved in pathways directly linked to proliferation and aggressiveness, including extracellular matrix organisation (*FN1*, *COL1A1*) [31], cytoskeletal regulation (*RHOA*) [32] and cell cycle control (*CDKN1A*, *CDC25B*, *CDCA4*, *RRM2*) [33,34]. These observations are consistent with the phenotypic effects observed across osteosarcoma cell lines and strengthen the link between miRNA-mediated regulation and functional outcomes. In addition, targets involved in transcriptional regulation (*SP1*, *SMARCA4*, *NCOR2*, *HDAC7*, *HMGA1*, *MIER2*) [35,36] and metabolic processes (*ALDOA*, *GALE*, *SLC25A23*, *TMEM135*, *ATG16L1*) align with the pathway-level changes identified in the integrative analysis, supporting a coordinated modulation of cellular homeostasis [37,38]. Importantly, these predicted miRNA–target interactions provide a direct link between OB-EVs molecular cargo and the modulation of proliferation and aggressiveness observed in osteosarcoma cells, supporting the functional relevance of the identified transcriptional changes.

Overall, the integration of transcriptomic and miRNA data points toward Wnt/β-catenin signalling as a relevant and convergent pathway potentially modulated by OB-EVs, although further functional validation will be required to confirm this mechanism.

Looking forward, our data open several avenues for future investigation. The observed modulation of key signalling pathways suggests that OB-EVs act through coordinated regulatory mechanisms rather than single dominant effectors. Dissecting the relative contribution of EV-associated miRNAs and protein cargo through targeted functional approaches will be essential to define the upstream regulators and downstream consequences of EV-mediated signalling. A direct comparison of signalling pathway activity across osteosarcoma cell lines displaying distinct phenotypic responses to OB-EVs will represent an important next step to better connect pathway modulation with functional outcomes. Moreover, the modulation of genes involved in proteostasis, ribosomal biogenesis and transcriptional control raises the possibility that OB-EVs reshape osteosarcoma cell behaviour by altering fundamental cellular processes. Ultimately, a deeper understanding of the molecular determinants underlying OB-EV activity may uncover novel vulnerabilities in osteosarcoma cells, potentially contributing to the development of new therapeutic strategies.

## 5. Study Limitations

The first limitation of this study is the lack of validation using in vivo models or protein-based assays. The uniqueness of the setup, with mouse OB-EVs used to target human cells, is also important to note as a limitation; nevertheless, the low immunogenicity, the efficacy and the biosafety of EVs in therapies have already been demonstrated in cross-species preclinical studies [39]. Notably, the RNAseq results were only partially reproduced using qRT-PCR. On one hand, it is expected that different cell lines, with distinct phenotypic and genetic backgrounds, will behave differently, and therefore it should not come as a surprise that U2OS and MG63 have only partially overlapping (sometimes opposite) transcriptional responses to OB-EVs treatment; on the other hand, MNNG/HOS results were also not completely reproduced. This may be due to several factors, including the different normalisation method (TPM vs. *GAPDH*), different sensitivity of the techniques, potential for false positives and artifacts due to the relatively low N for RNAseq and heterogeneity related to the nature of primary cell-derived material, as well as the limited 48 h treatment timepoint analysed. Moreover, despite our best efforts, primer design may be a source of variability when running larger panels of individually designed sets, especially with SYBR. Finally, while we focus our investigation on miRNAs as the “prime suspects”, it is important to note that other macromolecules may and most likely will play an important role in determining the observed phenotypes, which may be a target for future studies.

## 6. Conclusions

Overall, our findings highlight the ability of osteoblast-derived extracellular vesicles to modulate transcriptional and functional properties of osteosarcoma cells, at least in part through their miRNA cargo. The integration of transcriptomic and miRNA data supports the modulation of pathways associated with proliferation, cellular homeostasis and metabolic regulation. Among these, Wnt/β-catenin signalling emerged as a potentially relevant pathway, although this observation remains based on integrative and predictive analyses and will require further functional validation.

More broadly, these results provide a descriptive and hypothesis-generating framework that may help guide future mechanistic studies aimed at understanding the role of extracellular vesicles in osteosarcoma biology.

## Figures and Tables

**Table 1:** List of the top 20 downregulated transcripts in OB-EVs-treated MNNG/HOS versus untreated cells.

Gene Symbol	EntrezGeneID	|Fold Change|	*p*-Value
NMT1	4836	4.68 × 10^−4^	2.07 × 10^−3^
RPL17	6139	3.80 × 10^−3^	5.17 × 10^−3^
UBA52	7311	5.25 × 10^−3^	2.85 × 10^−2^
PAICS	10606	7.41 × 10^−3^	4.15 × 10^−3^
UBALD2	283991	1.20 × 10^−2^	8.77 × 10^−4^
NCOR2	9612	1.62 × 10^−2^	1.39 × 10^−3^
TSEN34	79042	2.29 × 10^−2^	1.99 × 10^−4^
ACTN1	87	2.45 × 10^−2^	1.12 × 10^−5^
EEF1D	1936	2.75 × 10^−2^	8.44 × 10^−3^
CDC34	997	3.31 × 10^−2^	4.61 × 10^−3^
SNRNP70	6625	3.89 × 10^−2^	4.23 × 10^−2^
PSMC3	5702	4.47 × 10^−2^	1.71 × 10^−2^
PTCD3	55037	4.47 × 10^−2^	5.95 × 10^−3^
CDKN1A	1026	4.90 × 10^−2^	2.31 × 10^−2^
KIFC3	3801	5.50 × 10^−2^	3.59 × 10^−2^
KLHL21	9903	5.62 × 10^−2^	1.56 × 10^−3^
NTAQ1	55093	6.92 × 10^−2^	2.27 × 10^−3^
S100A16	140576	7.08 × 10^−2^	1.43 × 10^−5^
ALDOA	226	7.08 × 10^−2^	7.90 × 10^−3^
CMTM3	123920	7.24 × 10^−2^	2.36 × 10^−4^

**Table 2:** List of the top 20 upregulated transcripts in OB-EVs-treated MNNG/HOS vs untreated cells.

Gene Symbol	EntrezGeneID	|Fold Change|	*p*-Value
RNY1	6084	3.16 × 10^10^	1.42 × 10^−12^
RPL36	25873	3.98 × 10^3^	4.14 × 10^−3^
EIF4A1	1973	2.57 × 10^2^	9.00 × 10^−5^
WAC	51322	1.78 × 10^2^	5.26 × 10^−3^
LMNA	4000	1.48 × 10^2^	1.95 × 10^−6^
HNRNPH1	3187	8.71 × 10^1^	1.97 × 10^−8^
ACADVL	37	4.27 × 10^1^	1.05 × 10^−2^
HNRNPC	3183	4.07 × 10^1^	1.93 × 10^−6^
PPT1	5538	3.89 × 10^1^	2.02 × 10^−2^
STUB1	10273	2.88 × 10^1^	2.31 × 10^−2^
SNHG32	50854	2.45 × 10^1^	3.59 × 10^−3^
MKRN1	23608	2.29 × 10^1^	6.21 × 10^−4^
C11orf68	83638	2.24 × 10^1^	2.46 × 10^−4^
PSMD6	9861	2.14 × 10^1^	2.12 × 10^−2^
EIF2A	83939	2.00 × 10^1^	2.19 × 10^−4^
BOLA1	51027	1.95 × 10^1^	1.48 × 10^−2^
HLA-B	3106	1.95 × 10^1^	3.49 × 10^−3^
UPF2	26019	1.82 × 10^1^	1.31 × 10^−3^
PPP1R18	107987457	1.78 × 10^1^	1.56 × 10^−2^
RPL8	6132	1.58 × 10^1^	2.45 × 10^−4^

**Table 3:** miRNAs expressed in osteoblast-derived EVs.

	EVs		Medium	
Target	CT Mean	Ct SE	CT Mean	Ct SE
* ath-miR159a	23.852	1.088	19.240	0.073
let-7e-5p	23.738	0.173	–	–
let-7g-5p	25.016	0.390	–	–
miR-140-5p	24.379	0.387	–	–
miR-152-3p	24.662	0.177	–	–
miR-185-5p	27.472	0.189	–	–
miR-195-5p	27.445	0.390	–	–
miR-26a-5p	22.512	0.396	–	–
miR-29b-3p	23.311	0.768	–	–
miR-30b-5p	23.602	0.373	–	–
miR-30c-5p	23.910	0.427	–	–
miR-324-5p	26.354	0.391	–	–
miR-34a-5p	22.985	0.913	–	–
miR-365a/b-3p	24.384	0.463	–	–

* spike-in miRNA used as internal control for miRNA purification.

**Table 4:** OB-EVs-expressed miRNA targets downregulated in OB-EVs-treated MNNG/HOS cells. Evaluation conducted in the databases specified in the header.

miRNA(s)	TargetScan	DIANA	miRDB	Common to 2+
miR-365a-3p/miR-365b-3p	*SP1*, *CMTM3*	*-*	*CMTM3*	*CMTM3*
miR-29b-3p	*TSPAN9*, *COL1A1*, *CD276*, *DEF8*, *DCTN1*, *ETV4*, *SP1*, *NCOR2*	*CD81*, *PPP2R1A*, *ATP6V1H*, *ARL4C*, *SLC4A2*, *CDCA4*, *ILK*, *PUF60*, *DHPS*, *GRK6*, *CD276*, *CDC25B*, *TSPO*, *SP1*, *FASN*, *FN1*, *TMEM135*, *NPTN*, *DDX5*, *CMTM3*, *CDKN1A*, *NCOR2*, *PAICS*, *RPL17*	*COL1A1*, *CDCA4*, *CD276*, *ETV4*, *SP1*, *CDKN1A*	*CDCA4*, *CD276*, *SP1*, *CDKN1A*, *COL1A1*, *ETV4*, *NCOR2*
miR-140-5p	*HDAC7, CAPN1*	*PFKL*, *PLXND1*, *PLK1*, *IER3*, *RAC2*, *EIF4G1*, *VASP*, *LAP3*, *LRP5*, *NT5E*, *DHPS*, *CKB*, *GPC1*, *EFHD2*, *DEF8*, *DCTN1*, *PKM*, *CS*, *MIER2*, *GALE*, *IGF2BP3*, *SMARCA4*, *ETV4*, *SEMA7A*, *ATG16L1*, *SP1*, *CALD1*, *UBASH3B*, *CAPN1*, *ITGB5*, *ANXA11*, *TPBG*, *NPTN*, *KIFC3*, *PTCD3*, *CDC34*	*HDAC7*, *CAPN1*	*CAPN1*, *HDAC7*
miR-152-3p	*CDC25B*, *ITGA1*, *U2AF1*, *UBASH3B*, *NPTN*	*TKT*, *PLXND1*, *PLK1*, *IER3*, *RAC2*, *BTBD2*, *VASP*, *PEMT*, *LAP3*, *LRP5*, *ILK*, *CKB*, *GPC1*, *EFHD2*, *CDC25B*, *COMT*, *PKM*, *SLC35A4*, *MIER2*, *GALE*, *ETV4*, *SP1*, *CALD1*, *UBASH3B*, *ITGB5*, *NT5DC2*, *CMC1*, *ANXA11*, *TPBG*, *NPTN*, *ATL2*, *KIFC3*, *CDC34*, *NMT1*	*BTBD2*, *TSPO*, *CS*, *NPTN*	*BTBD2*, *CDC25B*, *UBASH3B*, *NPTN*
miR-34a-5p	*ELOVL6*, *SLC25A23*, *U2AF1*, *ALDOA*	*CD81*, *PFKL*, *PPP2R1A*, *PLK1*, *RAC2*, *EIF4G1*, *LY6E*, *TSPO*, *MCM5*, *CALD1*, *PPP1R18*, *TMEM135*, *NPTN*, *ATL2*, *CMTM3*, *ALDOA*, *CDKN1A*, *PAICS*	*AGTRAP*, *SLC25A23*, *MIER2*, *LMNA*, *CMC1*, *ALDOA*	*ALDOA*, *SLC25A23*
miR-30b-5p/miR-30c-5p	*NT5E*, *MIER2*, *PPP1R18*, *ATL2*, *ACTN1*, *NCOR2*	*CD81*, *PPP2R1A*, *ARL4C*, *PLK1*, *EIF4G1*, *ILK*, *CDC25B*, *TSPO*, *SP1*, *CALD1*, *PPP1R18*, *TMEM135*, *NPTN*, *ATL2*, *DDX5*, *CMTM3*, *NCOR2*, *PAICS*, *RPL17*, *NMT1*	*ARL4C*, *NT5E*, *GRK6*, *MIER2*, *PPP1R18*, *PRPF19*, *OSBPL9*, *ATL2*	*ARL4C*, *NT5E*, *MIER2*, *PPP1R18*, *ATL2*, *NCOR2*
miR-26a-5p	*ARL4C*, *HMGA1*, *REEP4*	*CD81*, *ARL4C*, *IER3*, *RAC2*, *IGFBP2*, *VASP*, *ELOVL6*, *LRP5*, *SLC25A23*, *CS*, *ACSS2*, *SEMA7A*, *ATG16L1*, *REEP4*, *SP1*, *MFSD1*, *TMEM135*, *OSBPL9*, *DDX5*, *PAICS*	*ARL4C*, *HMGA1*, *ITPRID2*, *REEP4*, *TMEM135*	*ARL4C*, *REEP4*, *TMEM135*, *HMGA1*
let-7e-5p/let-7g-5p	*PLXND1*, *HMGA1*, *COL1A1*, *EFHD2*, *CD276*, *GALE*, *IGF2BP3*, *ATG16L1*, *MDFI*, *RRM2*, *CDC34*	*PFKL*, *PLXND1*, *PLK1*, *THOP1*, *BCAR1*, *IER3*, *RAC2*, *EIF4G1*, *VASP*, *LAP3*, *LRP5*, *NT5E*, *DHPS*, *CKB*, *GPC1*, *EFHD2*, *PKM*, *CS*, *MIER2*, *GALE*, *IGF2BP3*, *SMARCA4*, *ETV4*, *SEMA7A*, *ATG16L1*, *SP1*, *CALD1*, *UBASH3B*, *CAPN1*, *ITGB5*, *ANXA11*, *TPBG*, *KIFC3*, *PTCD3*, *CDC34*	*PLXND1*, *HMGA1*, *EFHD2*, *GALE*, *IGF2BP3*, *ATG16L1*, *MDFI*, *RRM2*, *ATL2*, *CDC34*	*PLXND1*, *EFHD2*, *GALE*, *IGF2BP3*, *ATG16L1*, *HMGA1*, *MDFI*, *RRM2*, *CDC34*
miR-195-5p	*GDI2*, *AGTRAP*, *PTGES2*, *ELOVL6*, *FLYWCH2*, *NUTF2*, *KRT81*, *LRPAP1*, *WLS*, *VPS41*, *DEF8*, *ACTG1*, *CD9*, *ITGA1*, *SAPCD2*, *SP1*, *PPP1R18*, *NPAS2*, *TMEM135*, *NME2*, *CMC1*, *TPBG*, *ALDOA*, *ACTN1*	*-*	*TMEM135*, *CMC1*	*TMEM135*, *CMC1*
miR-185-5p	*RHOA*, *CNIH2*, *TMEM135*	*TKT*, *PLXND1*, *PLK1*, *IER3*, *RAC2*, *BTBD2*, *EIF4G1*, *VASP*, *PEMT*, *LAP3*, *LRP5*, *ILK*, *CKB*, *GPC1*, *EFHD2*, *CDC25B*, *COMT*, *PKM*, *SLC35A4*, *MIER2*, *GALE*, *ETV4*, *SP1*, *CALD1*, *ITGB5*, *NT5DC2*, *FN1*, *CMC1*, *ANXA11*, *TPBG*, *NPTN*, *ATL2*, *KIFC3*, *CDC34*	*CLSTN1*, *HMGA1*, *KANK2*, *COL1A1*, *ACSS2*, *SAE1*, *RHOA*, *FN1*, *CNIH2*	*FN1*, *RHOA*, *CNIH2*
miR-324-5p	*SMARCA4*, *SP1*	-	*GPC1*, *ITGA1*, *SMARCA4*, *SP1*	*SMARCA4*, *SP1*

**Table 5:** Reactome description in common between the predicted OB-EVs miRNA target genes and the observed RNAseq data on MNNG/HOS treated with OB-EVs.

Reactome Description	*p*-Value (Predicted miRNA Targets)	*p*-Value (RNAseq)
PIP3 activates AKT signalling	1.93 × 10^−7^	8.61 × 10^−6^
Signalling by WNT	1.40 × 10^−15^	1.36 × 10^−4^
Transcriptional activity of SMAD2/SMAD3:SMAD4 heterotrimer	1.06 × 10^−7^	1.43 × 10^−4^
Regulation of mitotic cell cycle	3.02 × 10^−12^	2.10 × 10^−4^
TCF-dependent signalling in response to WNT	1.06 × 10^−10^	3.74 × 10^−4^
Deactivation of the beta-catenin transactivating complex	7.37 × 10^−8^	9.87 × 10^−4^
Beta-catenin-independent WNT signalling	9.08 × 10^−8^	1.50 × 10^−3^
Signalling by ERBB2	3.52 × 10^−10^	2.18 × 10^−3^
Signalling by FGFR2	2.08 × 10^−9^	2.22 × 10^−3^
SMAD2/SMAD3:SMAD4 heterotrimer regulates transcription	2.88 × 10^−6^	2.68 × 10^−3^
Oncogene Induced Senescence	5.16 × 10^−10^	3.01 × 10^−3^
G2/M Checkpoints	1.51 × 10^−10^	3.89 × 10^−3^
Downstream signalling events of B Cell Receptor (BCR)	6.81 × 10^−9^	4.22 × 10^−3^
Signalling by FGFR	2.08 × 10^−9^	5.32 × 10^−3^
Programmed Cell Death	2.55 × 10^−12^	5.50 × 10^−3^
Signalling by FGFR3	2.08 × 10^−9^	6.08 × 10^−3^
Apoptosis	2.47 × 10^−12^	6.09 × 10^−3^
Signalling by FGFR4	2.08 × 10^−9^	6.65 × 10^−3^
Cell Cycle Checkpoints	1.54 × 10^−9^	8.25 × 10^−3^
Signalling by EGFR in Cancer	5.94 × 10^−12^	1.05 × 10^−2^
Fc epsilon receptor (FCERI) signalling	1.92 × 10^−15^	1.19 × 10^−2^

## Data Availability

The original contributions presented in this study are included in the article/Supplementary Material. Further inquiries can be directed to the corresponding author.

## Supplementary Materials

The following supporting information can be downloaded at: https://www.mdpi.com/article/10.3390/biomedicines14051039/s1, Figure S1: Characterisation of osteoblast-derived EVs. Mouse primary osteoblasts were starved in serum-free DMEM for 24 h and EVs were isolated from conditioned medium (CM) by ultracentrifugation. (A) Size and concentration determination of OB-EVs by nanoparticle tracking analysis (NanoSight NS300). (B) Western blot for the EV positive markers CD63, Annexin II (Anxa2) and CD81 in protein lysates (7 µg) extracted from OB-EVs. (C) Transmission Electron Microscopy (TEM) evaluation of OB-EVs (arrows), original magnification 34,000×; insets: higher magnification (92,000×) in different fields of the same EVs sample. Data are representative of 3 independent preparations; Figure S2: Principal component analysis and distribution of normalised gene expression counts in OB-EVs-treated MNNG/HOS. (A) Principal Component Analysis (PCA) plot showing the separation of samples based on gene expression profiles. Samples are grouped into CTRL (red) and OB-EV-treated (blue) conditions, with 95% confidence ellipses indicating variability within each group. The first two principal components (Dim1 and Dim2) explain 31.4% and 29.3% of the total variance, respectively, highlighting distinct clustering between conditions. (B) Boxplot depicting the distribution of log2-transformed normalised gene expression counts across individual samples. Each box represents a sample, with the central line indicating the median expression level. The interquartile range (IQR) reflects sample variability, while whiskers extend to 1.5 times the IQR, with outliers shown as individual points. The consistent distribution across samples confirms effective normalisation, ensuring comparability of gene expression levels between conditions; Figure S3: Gene-Set Enrichment Analysis (GSEA) of DEGs in OB-EVs-treated MNNG/HOS cells. Gene-Set Enrichment Analysis (GSEA) using WEB-based GEne SeT AnaLysis Toolkit (WebGestalt, http://www.webgestalt.org). Bar graphs of pathway analysis performed using (A) KEGG, (B) Panther and (C) Reactome databases. The top 10 enriched/depleted terms were plotted based on Normalised Enrichment Score (NES). The NES of top enriched (blue bars) and top depleted (orange bars) pathways in OB-EVs-treated MNNG/HOS compared to untreated MNNG/HOS are listed. Pathways with an FDR < 0.05 are represented with dark blue or orange bars; pathways with an FDR ≥ 0.05 are represented with light blue or orange bars; Figure S4: Overall survival and metastasis-free survival analysis of *RPL17* in OB-EVs-treated MNNG/HOS cells. (A,B) Kaplan–Meier curves of Overall Survival (OS) and Metastasis-Free Survival (MFS) in osteosarcoma patients based on the expression levels of *RPL17*, analysed using the R2: Genomics Analysis and Visualization Platform. In both plots, the x-axis represents follow-up time (months), while the y-axis indicates survival probability. Statistical significance (log-rank *p*-values) and expression distribution graphs indicate gene expression variability across samples; Table S1: Effect of osteoblast-derived-EVs on osteosarcoma cells’ phenotype and on normal osteoblast cells. Results related to MNNG/HOS are from Ponzetti et al. [10]. Data are presented as mean fold change compared to control ± SD of at least three independent experiments. * *p* < 0.05, ** *p* < 0.01, paired *t*-test; Table S2: All up- and down-regulated transcripts in OB-EVs-treated MNNG/HOS cells vs control with *p* < 0.01 and a logFC > |0.5|; Data S1: miRNA profiling in OB-EVs; Supplementary materials and methods: 1. Nanoparticle tracking analysis. Extracellular vesicles (EVs) were isolated from osteoblast conditioned medium (CM) (12 mL collected from one 175 cm^2^ flask, cell density 35,000 cells/cm^2^) and resuspended in 100 µL of nanofiltered DPBS. OB-EVs were diluted 1:100 and used for nanoparticle tracking analysis using nanosight NS300 NTA. Flow and camera gain were adjusted following the manufacturer’s guidelines based on the NS300 quality control parameters. For each biological replicate, five 60-s camera acquisitions were analysed. 2. Transmission electron microscopy. EVs isolated from OB CM (12 mL collected from one 175 cm^2^ flask, cell density 35,000 cells/cm^2^) were fixed in 2% glutaraldehyde for 30 min. Ten µL of OB-EVs were then placed on Formvar-coated grids and maintained for 20 min in a dry environment to allow them to attach to the surface. Grids were washed in distilled water and contrasted with 1% phosphotungstic acid (PTA) for 2 min, washed again in distilled water and air-dried overnight. Pictures were then taken with a Philips CM100 Transmission electron microscope (TEM), with PHURONA camera (Emsis), at 80 kV.
