# Supplementary material for: Effect of Osteoblast-Derived Extracellular Vesicles on Osteosarcoma Cells’ Transcriptional Profile: Role of Shuttled miRNAs
Source: Biomedicines. 2026 May 3;14(5):1039. doi: 10.3390/biomedicines14051039 (PMC13204194; doi:10.3390/biomedicines14051039)
Supplement: Supplementary file 1 [file biomedicines-14-01039-s001.zip › Supplementary Figures.pdf]

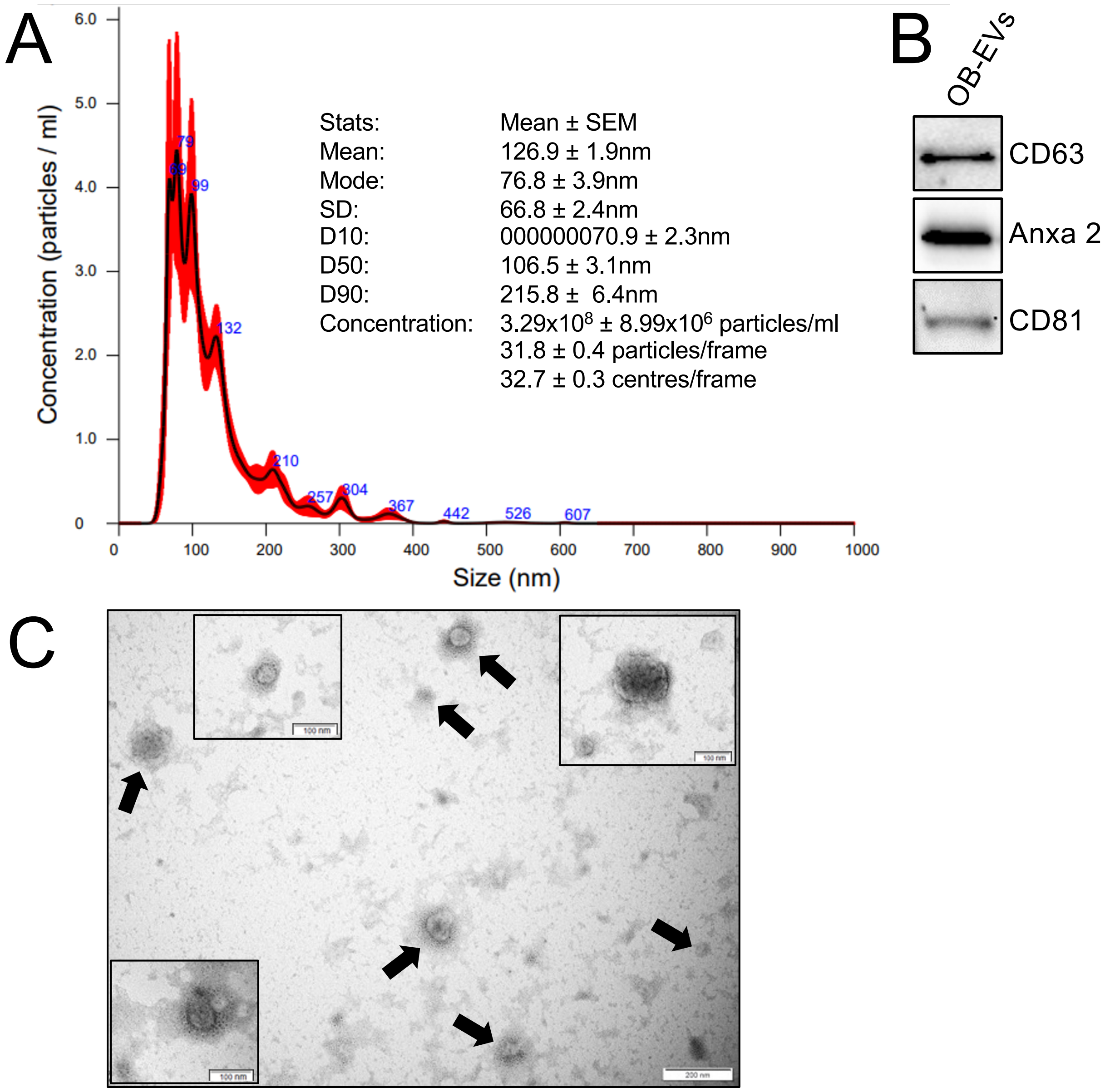

Figure S1. Characterisation of osteoblast-derived EVs. Mouse primary osteoblasts were starved in serum-free DMEM for 24 hours and EVs were isolated from conditioned medium (CM) by ultracentrifugation. **(A)** Size and concentration determination of OB-EVs by nanoparticle tracking analysis (NanoSight NS300). **(B)** Western blot for the EV positive markers CD63, Annexin II (Anxa2) and CD81 in protein lysates (7μg) extracted from OB-EVs. **(C)** Transmission Electron Microscopy (TEM) evaluation of OB-EVs (arrows), original magnification 34000x; insets: higher magnification (92000x) in different fields of the same EVs sample. Data are representative of 3 independent preparations.

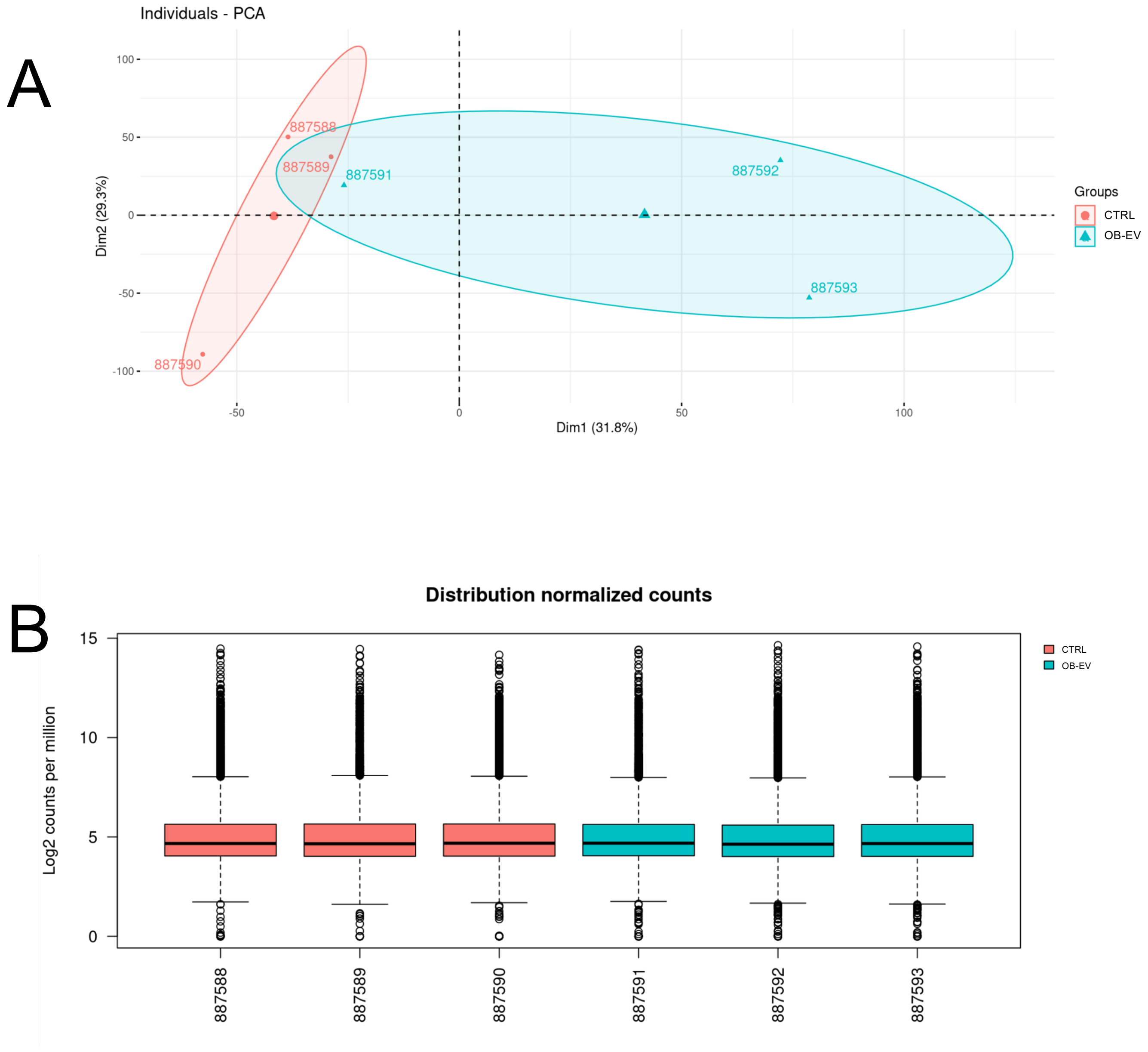

Figure S2. Principal component analysis and distribution of normalised gene expression counts in OB-EVs-treated MNNG/HOS. **(A)** Principal Component Analysis (PCA) plot showing the separation of samples based on gene expression profiles. Samples are grouped into CTRL (red) and OB-EV-treated (blue) conditions, with 95% confidence ellipses indicating variability within each group. The first two principal components (Dim1 and Dim2) explain 31.4% and 29.3% of the total variance, respectively, highlighting distinct clustering between conditions. **(B)** Boxplot depicting the distribution of log2-transformed normalised gene expression counts across individual samples. Each box represents a sample, with the central line indicating the median expression level. The interquartile range (IQR) reflects sample variability, while whiskers extend to 1.5 times the IQR, with outliers shown as individual points. The consistent distribution across samples confirms effective normalisation, ensuring comparability of gene expression levels between conditions.

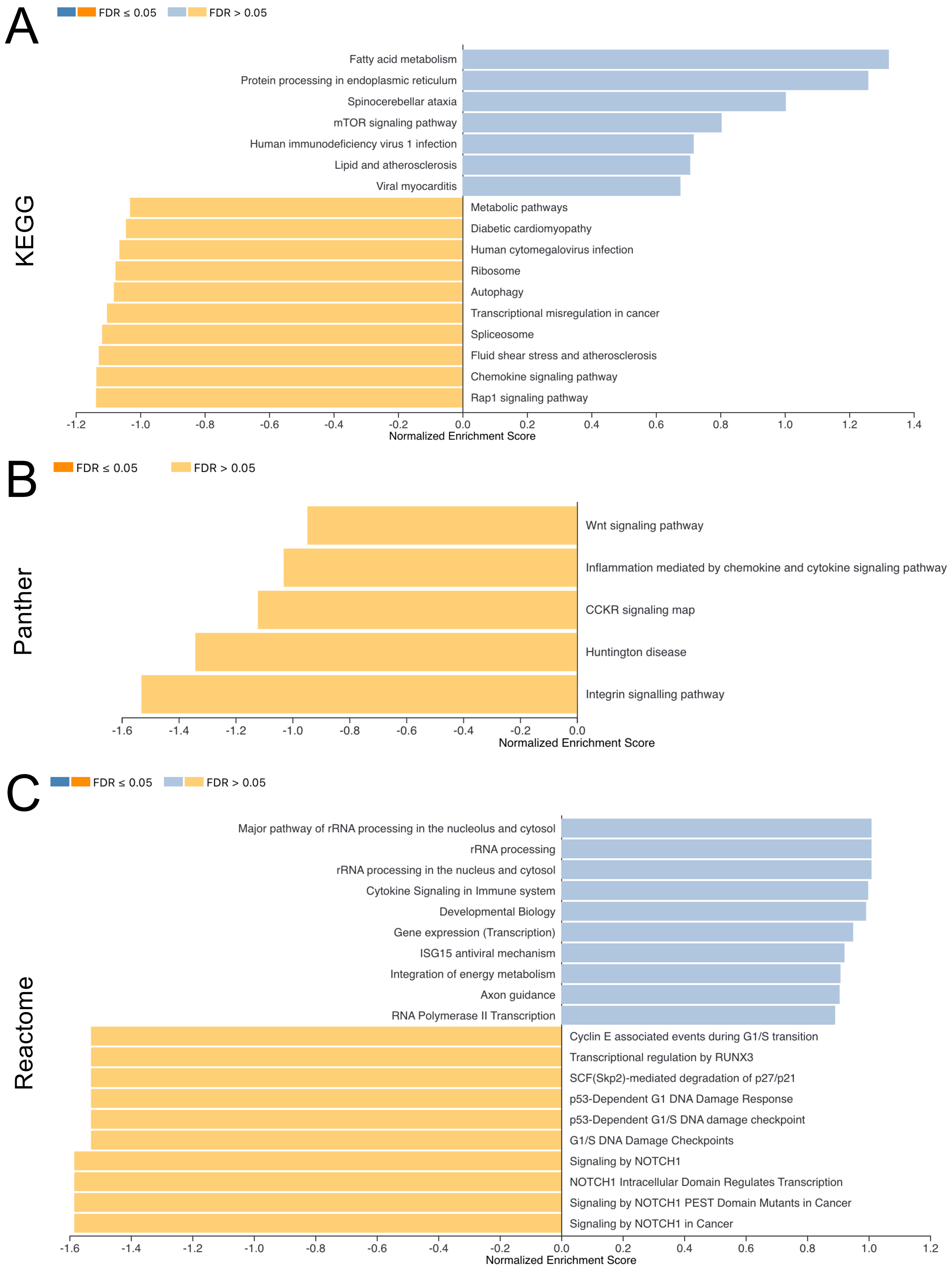

Figure S3. Gene-Set Enrichment Analysis (GSEA) of DEGs in OB-EVs-treated MNNG/HOS cells. Gene-Set Enrichment Analysis (GSEA) using WEB-based GEne SeT AnaLysis Toolkit (WebGestalt, http://www.webgestalt.org). Bar graphs of pathway analysis performed using **(A)** KEGG, **(B)** Panther and **(C)** Reactome databases. The top 10 enriched/depleted terms were plotted based on Normalized Enrichment Score (NES). The NES of top enriched (blue bars) and top depleted (orange bars) pathways in OB-EVs-treated MNNG/HOS compared to untreated MNNG/HOS are listed. Pathways with an FDR < 0.05 are represented with dark blue or orange bars; pathways with an FDR ≥ 0.05 are represented with light blue or orange bars.

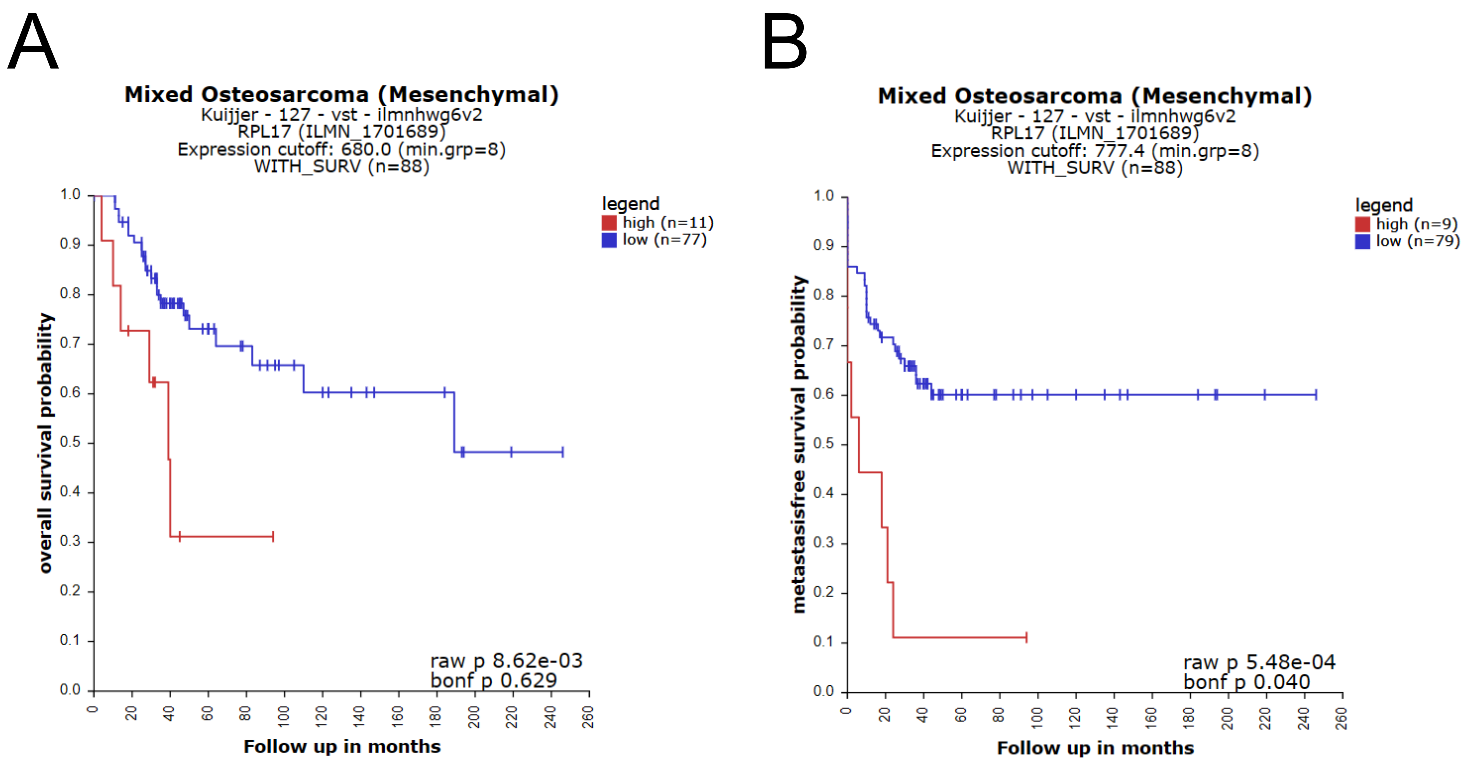

Figure S4. Overall survival and metastasis-free survival analysis of *RPL17* in OB-EVs-treated MNNG/HOS cells. (**A**,**B**) Kaplan–Meier curves of Overall Survival (OS) and Metastasis-Free Survival (MFS) in osteosarcoma patients based on the expression levels of *RPL17*, analysed using the R2: Genomics Analysis and Visualization Platform. In both plots, the x-axis represents follow-up time (months), while the y-axis indicates survival probability. Statistical significance (log-rank *p*-values) and expression distribution graphs indicate gene expression variability across samples.
