# Supplementary material for: Effect of Osteoblast-Derived Extracellular Vesicles on Osteosarcoma Cells’ Transcriptional Profile: Role of Shuttled miRNAs
Source: Biomedicines. 2026 May 3;14(5):1039. doi: 10.3390/biomedicines14051039 (PMC13204194; doi:10.3390/biomedicines14051039)
Supplement: Supplementary file 1 [file biomedicines-14-01039-s001.zip › Supplementary materials and methods.pdf]

1. Nanoparticle tracking analysis

Extracellular vesicles (EVs) were isolated from osteoblast conditioned medium (CM) (12 mL collected from one 175 cm^2 flask, cell density 35,000 cells/cm^2) and resuspended in 100 µL of nanofiltered DPBS. OB-EVs were diluted 1:100 and used for nanoparticle tracking analysis using nanosight NS300 NTA. Flow and camera gain were adjusted following the manufacturer's guidelines based on the NS300 quality control parameters. For each biological replicate, five 60-second camera acquisitions were analysed.

2. Transmission electron microscopy

EVs isolated from OB CM (12 mL collected from one 175 cm^2 flask, cell density 35,000 cells/cm^2) were fixed in 2% glutaraldehyde for 30 min. Ten µL of OB-EVs were then placed on Formvar-coated grids and maintained for 20 min in a dry environment to allow them to attach to the surface. Grids were washed in distilled water and contrasted with 1% phosphotungstic acid (PTA) for 2 min, washed again in distilled water and air-dried overnight. Pictures were then taken with a Philips CM100 Transmission electron microscope (TEM), with PHURONA camera (Emsis), at 80 kV.
