# Supplementary material for: Effect of Osteoblast-Derived Extracellular Vesicles on Osteosarcoma Cells’ Transcriptional Profile: Role of Shuttled miRNAs
Source: Biomedicines. 2026 May 3;14(5):1039. doi: 10.3390/biomedicines14051039 (PMC13204194; doi:10.3390/biomedicines14051039)
Supplement: Supplementary file 1 [file biomedicines-14-01039-s001.zip › Supplementary tables.pdf]

Table S1. Effect of osteoblast-derived-EVs on osteosarcoma cells' phenotype and on normal osteoblast cells. Results related to MNNG/HOS are from Ponzetti et al. [10]. Data are presented as mean fold change compared to control ± SD of at least three independent experiments. * p <0.05, ** p <0.01, paired t-test.

**MTT assay**							
**MNNG/HOS**		**U2OS**		**MG63**		**hFOB**	
Control	OB-EVs	Control	OB-EVs	Control	OB-EVs	Control	OB-EVs
1±0.221	0.609±0.177* * p <0.05	1±0.066	0.768±0.057* * p = 0.0137	1±0.034	0.902±0.123	1±0.269	0.959±0.199
**% Proliferating cells/field**							
**MNNG/HOS**		**U2OS**		**MG63**		**hFOB**	
Control	OB-EVs	Control	OB-EVs	Control	OB-EVs	Control	OB-EVs
1±0.039	0.840±0.081	1±0.057	0.587±0.051* *p = 0.017	1±0.369	4.927±1.283* *p = 0.020	1±0.161	1.048±0.270
**% Apoptotic cells**							
**MNNG/HOS**		**U2OS**		**MG63**		**hFOB**	
Control	OB-EVs	Control	OB-EVs	Control	OB-EVs	Control	OB-EVs
1±0.244	1.067±0.290	1±0.115	1.152±0.084* *p = 0.014	1±0.164	1.045±0.079	1±0.478	0.696±0.137
**N° Invading cells/field**							
**MNNG/HOS**		**U2OS**		**MG63**			
Control	OB-EVs	Control	OB-EVs	Control	OB-EVs		
1±0.462	0.659±0.322** **p < 0.01	1±0.334	1.152±0.509	1±0.345	1.261±0.540		

Table S2. All up- and down-regulated transcripts in OB-EVs-treated MNNG/HOS cells vs control with p <0.01 and a logFC >|0.5|

Transcript	Gene	Symbol	Entrezid	LogFC	FC	*P*-Value	FDR
ENST00000588975	ENSG00000136448	NMT1	4836	-3.33	$4.68 \cdot 10^{-4}$	$2.07 \cdot 10^{-3}$	$4.26 \cdot 10^{-1}$
ENST00000615479	ENSG00000265681	RPL17	6139	-2.42	$3.80 \cdot 10^{-3}$	$5.17 \cdot 10^{-3}$	$6.10 \cdot 10^{-1}$
ENST00000597451	ENSG00000221983	UBA52	7311	-2.28	$5.25 \cdot 10^{-3}$	$2.85 \cdot 10^{-2}$	1.00
ENST00000514888	ENSG00000128050	PAICS	10606	-2.13	$7.41 \cdot 10^{-3}$	$4.15 \cdot 10^{-3}$	$5.50 \cdot 10^{-1}$
ENST00000587913	ENSG00000185262	UBALD2	283991	-1.92	$1.20 \cdot 10^{-2}$	$8.77 \cdot 10^{-4}$	$2.96 \cdot 10^{-1}$
ENST00000536195	ENSG00000196498	NCOR2	9612	-1.79	$1.62 \cdot 10^{-2}$	$1.39 \cdot 10^{-3}$	$3.93 \cdot 10^{-1}$
ENST00000667261	ENSG00000170892	TSEN34	79042	-1.64	$2.29 \cdot 10^{-2}$	$1.99 \cdot 10^{-4}$	$1.61 \cdot 10^{-1}$
ENST00000555075	ENSG00000072110	ACTN1	87	-1.61	$2.45 \cdot 10^{-2}$	$1.12 \cdot 10^{-5}$	$2.08 \cdot 10^{-2}$
ENST00000531218	ENSG00000104529	EEF1D	1936	-1.56	$2.75 \cdot 10^{-2}$	$8.44 \cdot 10^{-3}$	$7.54 \cdot 10^{-1}$
ENST00000593036	ENSG00000099804	CDC34	997	-1.48	$3.31 \cdot 10^{-2}$	$4.61 \cdot 10^{-3}$	$5.87 \cdot 10^{-1}$
ENST00000597936	ENSG00000104852	SNRNP70	6625	-1.41	3.8910^{-2}	$4.23 \cdot 10^{-2}$	1.00
ENST00000602866	ENSG00000165916	PSMC3	5702	-1.35	$4.47 \cdot 10^{-2}$	$1.71 \cdot 10^{-2}$	$9.45 \cdot 10^{-1}$
ENST00000627371	ENSG00000132300	PTCD3	55037	-1.35	$4.47 \cdot 10^{-2}$	$5.95 \cdot 10^{-3}$	$6.41 \cdot 10^{-1}$

ENST00000244741	ENSG00000124762	CDKN1A	1026	-1.31	$4.90 \cdot 10^{-2}$	$2.31 \cdot 10^{-2}$	$9.99 \cdot 10^{-1}$
ENST00000445690	ENSG00000140859	KIFC3	3801	-1.26	$5.50 \cdot 10^{-2}$	$3.59 \cdot 10^{-2}$	1.00
ENST00000496707	ENSG00000162413	KLHL21	9903	-1.25	$5.62 \cdot 10^{-2}$	$1.56 \cdot 10^{-3}$	$3.93 \cdot 10^{-1}$
ENST00000522194	ENSG00000156795	NTAQ1	55093	-1.16	$6.92 \cdot 10^{-2}$	$2.27 \cdot 10^{-3}$	$4.36 \cdot 10^{-1}$
ENST00000368703	ENSG00000188643	S100A16	140576	-1.15	$7.08 \cdot 10^{-2}$	$1.43 \cdot 10^{-5}$	$2.27 \cdot 10^{-2}$
ENST00000566130	ENSG00000149925	ALDOA	226	-1.15	$7.08 \cdot 10^{-2}$	$7.90 \cdot 10^{-3}$	$7.26 \cdot 10^{-1}$
ENST00000564247	ENSG00000140931	CMTM3	123920	-1.14	$7.24 \cdot 10^{-2}$	$2.36 \cdot 10^{-4}$	$1.61 \cdot 10^{-1}$
ENST00000395633	ENSG00000099624	ATP5F1D	513	-1.13	$7.41 \cdot 10^{-2}$	$2.84 \cdot 10^{-3}$	$4.65 \cdot 10^{-1}$
ENST00000540698	ENSG00000108654	DDX5	1655	-1.11	$7.76 \cdot 10^{-2}$	$8.85 \cdot 10^{-5}$	$1.00 \cdot 10^{-1}$
ENST00000375136	ENSG00000158747	NBL1	100532736	-1.1	$7.94 \cdot 10^{-2}$	$3.58 \cdot 10^{-2}$	1.00
ENST00000567654	ENSG00000189091	SF3B3	23450	-1.09	$8.13 \cdot 10^{-2}$	$6.90 \cdot 10^{-3}$	$6.77 \cdot 10^{-1}$
ENST00000472097	ENSG00000119787	ATL2	64225	-1.08	$8.32 \cdot 10^{-2}$	$6.53 \cdot 10^{-3}$	$6.59 \cdot 10^{-1}$
ENST00000623691	ENSG00000169727	GPS1	2873	-1.04	$9.12 \cdot 10^{-2}$	$5.26 \cdot 10^{-3}$	$6.10 \cdot 10^{-1}$
ENST00000485717	ENSG00000171848	RRM2	6241	-1	$1.00 \cdot 10^{-1}$	$1.61 \cdot 10^{-2}$	$9.40 \cdot 10^{-1}$
ENST00000393190	ENSG00000243678	NME2	4831	-1	$1.00 \cdot 10^{-1}$	$8.34 \cdot 10^{-3}$	$7.54 \cdot 10^{-1}$
ENST00000504092	ENSG00000159692	CTBP1	1487	-0.98	$1.05 \cdot 10^{-1}$	$1.76 \cdot 10^{-2}$	$9.64 \cdot 10^{-1}$
ENST00000395914	ENSG00000087586	AURKA	6790	-0.98	$1.05 \cdot 10^{-1}$	$1.84 \cdot 10^{-2}$	$9.75 \cdot 10^{-1}$
ENST00000351217	ENSG00000156642	NPTN	27020	-0.97	$1.07 \cdot 10^{-1}$	$1.11 \cdot 10^{-2}$	$8.19 \cdot 10^{-1}$
ENST00000535040	ENSG00000146242	TPBG	7162	-0.97	$1.07 \cdot 10^{-1}$	$8.84 \cdot 10^{-3}$	$7.54 \cdot 10^{-1}$
ENST00000002501	ENSG00000003249	DBNDD1	79007	-0.96	$1.10 \cdot 10^{-1}$	$3.63 \cdot 10^{-3}$	$5.24 \cdot 10^{-1}$
ENST00000423894	ENSG00000187118	CMC1	152100	-0.95	$1.12 \cdot 10^{-1}$	$5.05 \cdot 10^{-4}$	$2.55 \cdot 10^{-1}$
ENST00000437799	ENSG00000122359	ANXA11	311	-0.95	$1.12 \cdot 10^{-1}$	$6.91 \cdot 10^{-3}$	$6.77 \cdot 10^{-1}$
ENST00000462759	ENSG00000117859	OSBPL9	114883	-0.95	$1.12 \cdot 10^{-1}$	$8.49 \cdot 10^{-3}$	$7.54 \cdot 10^{-1}$
ENST00000531800	ENSG00000166575	TMEM135	65084	-0.94	$1.15 \cdot 10^{-1}$	$7.94 \cdot 10^{-3}$	$7.26 \cdot 10^{-1}$
ENST00000503064	ENSG00000243678	NME2	4831	-0.94	$1.15 \cdot 10^{-1}$	$8.84 \cdot 10^{-3}$	$7.54 \cdot 10^{-1}$
ENST00000507868	ENSG00000113648	MACROH2A1	9555	-0.93	$1.17 \cdot 10^{-1}$	$9.02 \cdot 10^{-3}$	$7.54 \cdot 10^{-1}$
ENST00000311445	ENSG00000174871	CNIH2	254263	-0.92	$1.20 \cdot 10^{-1}$	$2.74 \cdot 10^{-2}$	1.00
ENST00000443816	ENSG00000115414	FN1	2335	-0.92	$1.20 \cdot 10^{-1}$	$6.93 \cdot 10^{-4}$	$2.67 \cdot 10^{-1}$
ENST00000306897	ENSG00000169750	RAC3	5881	-0.91	$1.23 \cdot 10^{-1}$	$2.46 \cdot 10^{-3}$	$4.56 \cdot 10^{-1}$
ENST00000431929	ENSG00000067560	RHOA	387	-0.91	$1.23 \cdot 10^{-1}$	$3.89 \cdot 10^{-2}$	1.00
ENST00000397817	ENSG00000103152	MPG	4350	-0.91	$1.23 \cdot 10^{-1}$	$7.94 \cdot 10^{-4}$	$2.76 \cdot 10^{-1}$

ENST00000355443	ENSG00000109736	MFSD10	10227	-0.9	$1.26 \cdot 10^{-1}$	$2.03 \cdot 10^{-3}$	$4.26 \cdot 10^{-1}$
ENST00000541371	ENSG00000110107	PRPF19	27339	-0.9	$1.26 \cdot 10^{-1}$	$3.88 \cdot 10^{-2}$	1.00
ENST00000412681	ENSG00000154146	NRGN	4900	-0.9	$1.26 \cdot 10^{-1}$	$4.64 \cdot 10^{-3}$	$5.87 \cdot 10^{-1}$
ENST00000469565	ENSG00000160789	LMNA	4000	-0.9	$1.26 \cdot 10^{-1}$	$7.66 \cdot 10^{-4}$	$2.75 \cdot 10^{-1}$
ENST00000355815	ENSG00000072310	SREBF1	6720	-0.89	$1.29 \cdot 10^{-1}$	$1.18 \cdot 10^{-2}$	$8.37 \cdot 10^{-1}$
ENST00000618619	ENSG00000265681	RPL17	6139	-0.89	$1.29 \cdot 10^{-1}$	$1.85 \cdot 10^{-2}$	$9.75 \cdot 10^{-1}$
ENST00000433408	ENSG00000170485	NPAS2	4862	-0.89	$1.29 \cdot 10^{-1}$	$2.06 \cdot 10^{-2}$	$9.75 \cdot 10^{-1}$
ENST00000395560	ENSG00000183696	UPP1	7378	-0.89	$1.29 \cdot 10^{-1}$	$2.28 \cdot 10^{-2}$	$9.98 \cdot 10^{-1}$
ENST00000463947	ENSG00000168268	NT5DC2	64943	-0.89	$1.29 \cdot 10^{-1}$	$3.86 \cdot 10^{-3}$	$5.35 \cdot 10^{-1}$
ENST00000563192	ENSG00000260260	SNHG19		-0.88	$1.32 \cdot 10^{-1}$	$1.03 \cdot 10^{-3}$	$3.37 \cdot 10^{-1}$
ENST00000481591	ENSG00000082781	ITGB5	3693	-0.88	$1.32 \cdot 10^{-1}$	$1.98 \cdot 10^{-2}$	$9.75 \cdot 10^{-1}$
ENST00000457918	ENSG00000143575	HAX1	10456	-0.88	$1.32 \cdot 10^{-1}$	$3.64 \cdot 10^{-2}$	1.00
ENST00000614502	ENSG00000120913	PDLIM2	64236	-0.88	$1.32 \cdot 10^{-1}$	$4.86 \cdot 10^{-2}$	1.00
ENST00000306749	ENSG00000169710	FASN	2194	-0.87	$1.35 \cdot 10^{-1}$	$1.39 \cdot 10^{-2}$	$8.96 \cdot 10^{-1}$
ENST00000279247	ENSG00000014216	CAPN1	823	-0.87	$1.35 \cdot 10^{-1}$	$2.49 \cdot 10^{-2}$	1.00
ENST00000399199	ENSG00000146112	PPP1R18	107987457	-0.87	$1.35 \cdot 10^{-1}$	$9.64 \cdot 10^{-3}$	$7.82 \cdot 10^{-1}$
ENST00000530917	ENSG00000154127	UBASH3B	84959	-0.86	$1.38 \cdot 10^{-1}$	$1.40 \cdot 10^{-2}$	$8.97 \cdot 10^{-1}$
ENST00000393118	ENSG00000122786	CALD1	800	-0.85	$1.41 \cdot 10^{-1}$	$2.06 \cdot 10^{-2}$	$9.75 \cdot 10^{-1}$
ENST00000423161	ENSG00000072310	SREBF1	6720	-0.85	$1.41 \cdot 10^{-1}$	$5.69 \cdot 10^{-4}$	$2.56 \cdot 10^{-1}$
ENST00000432027	ENSG00000112559	MDFI	4188	-0.84	$1.45 \cdot 10^{-1}$	$2.76 \cdot 10^{-3}$	$4.65 \cdot 10^{-1}$
ENST00000476803	ENSG00000162585	FAAP20	199990	-0.84	$1.45 \cdot 10^{-1}$	$3.89 \cdot 10^{-3}$	$5.35 \cdot 10^{-1}$
ENST00000415822	ENSG00000118855	MFSD1	64747	-0.84	$1.45 \cdot 10^{-1}$	$5.42 \cdot 10^{-4}$	$2.56 \cdot 10^{-1}$
ENST00000270225	ENSG00000142230	SAE1	10055	-0.83	$1.48 \cdot 10^{-1}$	$2.04 \cdot 10^{-2}$	$9.75 \cdot 10^{-1}$
ENST00000392017	ENSG00000085978	ATG16L1	55054	-0.83	$1.48 \cdot 10^{-1}$	$2.45 \cdot 10^{-2}$	1.00
ENST00000306306	ENSG00000168476	REEP4	80346	-0.83	$1.48 \cdot 10^{-1}$	$2.55 \cdot 10^{-2}$	1.00
ENST00000426431	ENSG00000185591	SP1	6667	-0.83	$1.48 \cdot 10^{-1}$	$2.72 \cdot 10^{-2}$	1.00
ENST00000372469	ENSG00000167157	PRRX2	51450	-0.81	$1.55 \cdot 10^{-1}$	$1.25 \cdot 10^{-2}$	$8.50 \cdot 10^{-1}$
ENST00000261918	ENSG00000138623	SEMA7A	8482	-0.81	$1.55 \cdot 10^{-1}$	$2.67 \cdot 10^{-2}$	1.00
ENST00000393664	ENSG00000175832	ETV4	2118	-0.8	$1.58 \cdot 10^{-1}$	$1.08 \cdot 10^{-2}$	$8.12 \cdot 10^{-1}$
ENST00000233627	ENSG00000115286	NDUFS7	374291	-0.8	$1.58 \cdot 10^{-1}$	$2.24 \cdot 10^{-2}$	$9.88 \cdot 10^{-1}$
ENST00000368400	ENSG00000179085	DPM3	54344	-0.8	$1.58 \cdot 10^{-1}$	$2.34 \cdot 10^{-4}$	$1.61 \cdot 10^{-1}$

ENST00000341197	ENSG00000124191	TOX2	84969	-0.8	$1.58 \cdot 10^{-1}$	$7.62 \cdot 10^{-4}$	$2.75 \cdot 10^{-1}$
ENST00000521007	ENSG00000064313	TAF2	6873	-0.79	$1.62 \cdot 10^{-1}$	$2.42 \cdot 10^{-2}$	1.00
ENST00000617571	ENSG00000203485	INF2	64423	-0.78	$1.66 \cdot 10^{-1}$	$1.25 \cdot 10^{-2}$	$8.50 \cdot 10^{-1}$
ENST00000476627	ENSG00000164897	TMUB1	83590	-0.78	$1.66 \cdot 10^{-1}$	$1.96 \cdot 10^{-3}$	$4.19 \cdot 10^{-1}$
ENST00000444061	ENSG00000127616	SMARCA4	6597	-0.78	$1.66 \cdot 10^{-1}$	$3.87 \cdot 10^{-2}$	1.00
ENST00000380276	ENSG00000160201	U2AF1	7307	-0.78	$1.66 \cdot 10^{-1}$	$5.90 \cdot 10^{-3}$	$6.41 \cdot 10^{-1}$
ENST00000635755	ENSG00000105556	MIER2	54531	-0.77	$1.70 \cdot 10^{-1}$	$1.79 \cdot 10^{-2}$	$9.67 \cdot 10^{-1}$
ENST00000521605	ENSG00000147586	MRPS28	28957	-0.77	$1.70 \cdot 10^{-1}$	$1.79 \cdot 10^{-2}$	$9.67 \cdot 10^{-1}$
ENST00000617979	ENSG00000117308	GALE	2582	-0.77	$1.70 \cdot 10^{-1}$	$1.99 \cdot 10^{-2}$	$9.75 \cdot 10^{-1}$
ENST00000480547	ENSG00000136231	IGF2BP3	10643	-0.77	$1.70 \cdot 10^{-1}$	$2.39 \cdot 10^{-2}$	1.00
ENST00000538833	ENSG00000169718	DUS1L	64118	-0.77	$1.70 \cdot 10^{-1}$	$3.86 \cdot 10^{-2}$	1.00
ENST00000409687	ENSG00000186193	SAPCD2	89958	-0.76	$1.74 \cdot 10^{-1}$	$1.90 \cdot 10^{-2}$	$9.75 \cdot 10^{-1}$
ENST00000397375	ENSG00000074071	MRPS34	65993	-0.76	$1.74 \cdot 10^{-1}$	$2.25 \cdot 10^{-2}$	$9.88 \cdot 10^{-1}$
ENST00000219548	ENSG00000103266	STUB1	10273	-0.76	$1.74 \cdot 10^{-1}$	$2.57 \cdot 10^{-2}$	1.00
ENST00000453943	ENSG00000143774	GUK1	2987	-0.76	$1.74 \cdot 10^{-1}$	$9.58 \cdot 10^{-3}$	$7.82 \cdot 10^{-1}$
ENST00000327490	ENSG00000185262	UBALD2	283991	-0.75	$1.78 \cdot 10^{-1}$	$1.51 \cdot 10^{-3}$	$3.93 \cdot 10^{-1}$
ENST00000650673	ENSG00000213949	ITGA1	3672	-0.75	$1.78 \cdot 10^{-1}$	$1.93 \cdot 10^{-2}$	$9.75 \cdot 10^{-1}$
ENST00000279281	ENSG00000149823	VPS51	738	-0.75	$1.78 \cdot 10^{-1}$	$2.15 \cdot 10^{-2}$	$9.79 \cdot 10^{-1}$
ENST00000530826	ENSG00000154027	AK5	26289	-0.75	$1.78 \cdot 10^{-1}$	$3.45 \cdot 10^{-2}$	1.00
ENST00000360596	ENSG00000131069	ACSS2	55902	-0.75	$1.78 \cdot 10^{-1}$	$4.92 \cdot 10^{-2}$	1.00
ENST00000548849	ENSG00000062485	CS	1431	-0.74	$1.82 \cdot 10^{-1}$	$1.69 \cdot 10^{-2}$	$9.45 \cdot 10^{-1}$
ENST00000612662	ENSG00000176087	SLC35A4	113829	-0.74	$1.82 \cdot 10^{-1}$	$2.63 \cdot 10^{-3}$	$4.57 \cdot 10^{-1}$
ENST00000382519	ENSG00000010278	CD9	928	-0.74	$1.82 \cdot 10^{-1}$	$3.09 \cdot 10^{-2}$	1.00
ENST00000382011	ENSG00000100297	MCM5	4174	-0.74	$1.82 \cdot 10^{-1}$	$3.65 \cdot 10^{-2}$	1.00
ENST00000561609	ENSG00000067225	PKM	5315	-0.73	$1.86 \cdot 10^{-1}$	$1.04 \cdot 10^{-2}$	$8.12 \cdot 10^{-1}$
ENST00000448866	ENSG00000059377	TBXAS1	6916	-0.73	$1.86 \cdot 10^{-1}$	$3.54 \cdot 10^{-2}$	1.00
ENST00000591871	ENSG00000099800	TIMM13	26517	-0.72	$1.91 \cdot 10^{-1}$	$2.76 \cdot 10^{-4}$	$1.71 \cdot 10^{-1}$
ENST00000585479	ENSG00000105355	PLIN3	10226	-0.72	$1.91 \cdot 10^{-1}$	$3.97 \cdot 10^{-2}$	1.00
ENST00000412786	ENSG00000093010	COMT	1312	-0.71	$1.95 \cdot 10^{-1}$	$1.24 \cdot 10^{-2}$	$8.50 \cdot 10^{-1}$
ENST00000373422	ENSG00000134686	PHC2	1912	-0.71	$1.95 \cdot 10^{-1}$	$5.38 \cdot 10^{-3}$	$6.16 \cdot 10^{-1}$
ENST00000583777	ENSG00000100300	TSPO	706	-0.7	$2.00 \cdot 10^{-1}$	$2.95 \cdot 10^{-2}$	1.00

ENST00000570382	ENSG00000184009	ACTG1	71	-0.69	$2.04·10^{-1}$	$1.08·10^{-2}$	$8.12·10^{-1}$
ENST00000409240	ENSG00000204843	DCTN1	1639	-0.69	$2.04·10^{-1}$	$1.66·10^{-3}$	$3.93·10^{-1}$
ENST00000533397	ENSG00000161016	RPL8	6132	-0.69	$2.04·10^{-1}$	$3.60·10^{-2}$	1.00
ENST00000610455	ENSG00000140995	DEF8	54849	-0.68	$2.09·10^{-1}$	$2.32·10^{-3}$	$4.37·10^{-1}$
ENST00000557094	ENSG00000140105	WARS1	7453	-0.68	$2.09·10^{-1}$	$3.33·10^{-2}$	1.00
ENST00000262965	ENSG00000071564	TCF3	6929	-0.68	$2.09·10^{-1}$	$4.57·10^{-2}$	1.00
ENST00000326294	ENSG00000213402	PTPRCAP	5790	-0.67	$2.14·10^{-1}$	$2.07·10^{-2}$	$9.75·10^{-1}$
ENST00000371696	ENSG00000169692	AGPAT2	10555	-0.67	$2.14·10^{-1}$	$2.88·10^{-3}$	$4.65·10^{-1}$
ENST00000245960	ENSG00000101224	CDC25B	994	-0.66	$2.19·10^{-1}$	$2.12·10^{-2}$	$9.75·10^{-1}$
ENST00000426960	ENSG00000182196	ARL6IP4	51329	-0.66	$2.19·10^{-1}$	$3.61·10^{-3}$	$5.24·10^{-1}$
ENST00000309032	ENSG00000002330	BAD	572	-0.66	$2.19·10^{-1}$	$3.77·10^{-2}$	1.00
ENST00000354334	ENSG00000061273	HDAC7	51564	-0.66	$2.19·10^{-1}$	$4.05·10^{-2}$	1.00
ENST00000484600	ENSG00000130429	ARPC1B	10095	-0.66	$2.19·10^{-1}$	$5.06·10^{-6}$	$1.13·10^{-2}$
ENST00000414632	ENSG00000006715	VPS41	27072	-0.65	$2.24·10^{-1}$	$3.56·10^{-2}$	1.00
ENST00000528610	ENSG00000104529	EEF1D	1936	-0.64	$2.29·10^{-1}$	$1.12·10^{-2}$	$8.19·10^{-1}$
ENST00000318443	ENSG00000103855	CD276	80381	-0.64	$2.29·10^{-1}$	$1.69·10^{-2}$	$9.45·10^{-1}$
ENST00000597529	ENSG00000142227	EMP3	2014	-0.64	$2.29·10^{-1}$	$2.80·10^{-2}$	1.00
ENST00000354777	ENSG00000116729	WLS	79971	-0.64	$2.29·10^{-1}$	$3.24·10^{-2}$	1.00
ENST00000374610	ENSG00000204220	PFDN6	10471	-0.63	$2.34·10^{-1}$	$1.78·10^{-2}$	$9.67·10^{-1}$
ENST00000266263	ENSG00000242114	MTFP1	51537	-0.63	$2.34·10^{-1}$	$1.98·10^{-2}$	$9.75·10^{-1}$
ENST00000361157	ENSG00000126709	IFI6	2537	-0.63	$2.34·10^{-1}$	$3.36·10^{-2}$	1.00
ENST00000403346	ENSG00000115694	STK25	10494	-0.63	$2.34·10^{-1}$	$5.93·10^{-3}$	$6.41·10^{-1}$
ENST00000264039	ENSG00000063660	GPC1	2817	-0.62	$2.40·10^{-1}$	$1.34·10^{-2}$	$8.71·10^{-1}$
ENST00000375980	ENSG00000142634	EFHD2	79180	-0.62	$2.40·10^{-1}$	$1.52·10^{-2}$	$9.33·10^{-1}$
ENST00000220772	ENSG00000104332	SFRP1	6422	-0.62	$2.40·10^{-1}$	$2.18·10^{-3}$	$4.34·10^{-1}$
ENST00000357429	ENSG00000146540	C7orf50	84310	-0.62	$2.40·10^{-1}$	$2.57·10^{-2}$	1.00
ENST00000440747	ENSG00000164818	DNAAF5	54919	-0.62	$2.40·10^{-1}$	$3.59·10^{-2}$	1.00
ENST00000296325	ENSG00000163956	LRPAP1	4043	-0.62	$2.40·10^{-1}$	$6.11·10^{-3}$	$6.47·10^{-1}$
ENST00000429120	ENSG00000160932	LY6E	4061	-0.61	$2.45·10^{-1}$	$1.14·10^{-2}$	$8.31·10^{-1}$
ENST00000526683	ENSG00000179950	PUF60	22827	-0.61	$2.45·10^{-1}$	$1.92·10^{-3}$	$4.19·10^{-1}$
ENST00000354464	ENSG00000196497	IPO4	79711	-0.61	$2.45·10^{-1}$	$3.48·10^{-2}$	1.00

ENST00000372715	ENSG00000119333	DYNC2I2	89891	-0.61	$2.45\cdot10^{-1}$	$3.66\cdot10^{-2}$	1.00
ENST00000348956	ENSG00000166165	CKB	1152	-0.61	$2.45\cdot10^{-1}$	$4.32\cdot10^{-2}$	1.00
ENST00000245615	ENSG00000125505	MBOAT7	79143	-0.61	$2.45\cdot10^{-1}$	$4.52\cdot10^{-2}$	1.00
ENST00000210060	ENSG00000095059	DHPS	1725	-0.61	$2.45\cdot10^{-1}$	$5.74\cdot10^{-3}$	$6.41\cdot10^{-1}$
ENST00000528793	ENSG00000198055	GRK6	2870	-0.61	$2.45\cdot10^{-1}$	$9.09\cdot10^{-3}$	$7.54\cdot10^{-1}$
ENST00000375635	ENSG00000204387	SNHG32	50854	-0.6	$2.51\cdot10^{-1}$	$1.27\cdot10^{-2}$	$8.53\cdot10^{-1}$
ENST00000294304	ENSG00000162337	LRP5	4041	-0.6	$2.51\cdot10^{-1}$	$1.54\cdot10^{-4}$	$1.55\cdot10^{-1}$
ENST00000420936	ENSG00000166333	ILK	3611	-0.6	$2.51\cdot10^{-1}$	$2.04\cdot10^{-2}$	$9.75\cdot10^{-1}$
ENST00000327741	ENSG00000205426	KRT81	3887	-0.6	$2.51\cdot10^{-1}$	$2.75\cdot10^{-2}$	1.00
ENST00000301454	ENSG00000125648	SLC25A23	79085	-0.6	$2.51\cdot10^{-1}$	$3.09\cdot10^{-2}$	1.00
ENST00000257770	ENSG00000135318	NT5E	4907	-0.6	$2.51\cdot10^{-1}$	$6.18\cdot10^{-4}$	$2.56\cdot10^{-1}$
ENST00000225964	ENSG00000108821	COL1A1	1277	-0.59	$2.57\cdot10^{-1}$	$1.29\cdot10^{-2}$	$8.56\cdot10^{-1}$
ENST00000618908	ENSG00000002549	LAP3	51056	-0.59	$2.57\cdot10^{-1}$	$2.50\cdot10^{-2}$	1.00
ENST00000336219	ENSG00000170779	CDCA4	55038	-0.59	$2.57\cdot10^{-1}$	$3.57\cdot10^{-2}$	1.00
ENST00000354724	ENSG00000164683	HEY1	23462	-0.59	$2.57\cdot10^{-1}$	$4.40\cdot10^{-2}$	1.00
ENST00000568233	ENSG00000102898	NUTF2	10204	-0.58	$2.63\cdot10^{-1}$	$1.94\cdot10^{-2}$	$9.75\cdot10^{-1}$
ENST00000586659	ENSG00000197256	KANK2	25959	-0.58	$2.63\cdot10^{-1}$	$2.40\cdot10^{-2}$	1.00
ENST00000422725	ENSG00000228594	FNDC10	643988	-0.58	$2.63\cdot10^{-1}$	$3.06\cdot10^{-2}$	1.00
ENST00000417626	ENSG00000214706	IFRD2	7866	-0.58	$2.63\cdot10^{-1}$	$3.30\cdot10^{-2}$	1.00
ENST00000255389	ENSG00000133027	PEMT	10400	-0.58	$2.63\cdot10^{-1}$	$4.14\cdot10^{-3}$	$5.50\cdot10^{-1}$
ENST00000488034	ENSG00000112514	CUTA	51596	-0.57	$2.69\cdot10^{-1}$	$1.88\cdot10^{-2}$	$9.75\cdot10^{-1}$
ENST00000381519	ENSG00000214026	MRPL23	107987373	-0.57	$2.69\cdot10^{-1}$	$1.90\cdot10^{-3}$	$4.19\cdot10^{-1}$
ENST00000369409	ENSG00000092621	PHGDH	26227	-0.57	$2.69\cdot10^{-1}$	$2.09\cdot10^{-2}$	$9.75\cdot10^{-1}$
ENST00000409001	ENSG00000138434	ITPRID2	6744	-0.57	$2.69\cdot10^{-1}$	$4.36\cdot10^{-3}$	$5.70\cdot10^{-1}$
ENST00000340611	ENSG00000106009	BRAT1	221927	-0.57	$2.69\cdot10^{-1}$	$4.96\cdot10^{-2}$	1.00
ENST00000413384	ENSG00000164889	SLC4A2	6522	-0.56	$2.75\cdot10^{-1}$	$1.52\cdot10^{-3}$	$3.93\cdot10^{-1}$
ENST00000255608	ENSG00000133243	BTBD2	55643	-0.56	$2.75\cdot10^{-1}$	$1.64\cdot10^{-2}$	$9.44\cdot10^{-1}$
ENST00000249071	ENSG00000128340	RAC2	5880	-0.56	$2.75\cdot10^{-1}$	$1.65\cdot10^{-3}$	$3.93\cdot10^{-1}$
ENST00000215587	ENSG00000099817	POLR2E	5434	-0.56	$2.75\cdot10^{-1}$	$2.23\cdot10^{-2}$	$9.88\cdot10^{-1}$
ENST00000375663	ENSG00000204394	VARS1	7407	-0.56	$2.75\cdot10^{-1}$	$2.99\cdot10^{-3}$	$4.68\cdot10^{-1}$
ENST00000496756	ENSG00000092621	PHGDH	26227	-0.56	$2.75\cdot10^{-1}$	$3.37\cdot10^{-3}$	$5.21\cdot10^{-1}$

ENST00000346169	ENSG00000114867	EIF4G1	1981	-0.56	$2.75 \cdot 10^{-1}$	$3.82 \cdot 10^{-2}$	1.00
ENST00000233809	ENSG00000115457	IGFBP2	3485	-0.56	$2.75 \cdot 10^{-1}$	$4.35 \cdot 10^{-2}$	1.00
ENST00000245932	ENSG00000125753	VASP	7408	-0.56	$2.75 \cdot 10^{-1}$	$4.67 \cdot 10^{-2}$	1.00
ENST00000302274	ENSG00000170522	ELOVL6	79071	-0.56	$2.75 \cdot 10^{-1}$	$4.69 \cdot 10^{-2}$	1.00
ENST00000396958	ENSG00000162076	FLYWCH2	114984	-0.56	$2.75 \cdot 10^{-1}$	$4.93 \cdot 10^{-2}$	1.00
ENST00000311487	ENSG00000137309	HMGA1	3159	-0.56	$2.75 \cdot 10^{-1}$	$6.02 \cdot 10^{-4}$	$2.56 \cdot 10^{-1}$
ENST00000420641	ENSG00000050820	BCAR1	9564	-0.55	$2.82 \cdot 10^{-1}$	$1.58 \cdot 10^{-2}$	$9.38 \cdot 10^{-1}$
ENST00000338961	ENSG00000148334	PTGES2	80142	-0.55	$2.82 \cdot 10^{-1}$	$1.61 \cdot 10^{-2}$	$9.40 \cdot 10^{-1}$
ENST00000259874	ENSG00000137331	IER3	8870	-0.55	$2.82 \cdot 10^{-1}$	$2.23 \cdot 10^{-2}$	$9.88 \cdot 10^{-1}$
ENST00000011898	ENSG00000011105	TSPAN9	10867	-0.55	$2.82 \cdot 10^{-1}$	$2.50 \cdot 10^{-2}$	1.00
ENST00000300093	ENSG00000166851	PLK1	5347	-0.55	$2.82 \cdot 10^{-1}$	$2.82 \cdot 10^{-3}$	$4.65 \cdot 10^{-1}$
ENST00000586677	ENSG00000172009	THOP1	7064	-0.55	$2.82 \cdot 10^{-1}$	$3.20 \cdot 10^{-2}$	1.00
ENST00000394225	ENSG00000109390	NDUFC1	4717	-0.55	$2.82 \cdot 10^{-1}$	$3.87 \cdot 10^{-2}$	1.00
ENST00000361311	ENSG00000171603	CLSTN1	22883	-0.55	$2.82 \cdot 10^{-1}$	$4.94 \cdot 10^{-3}$	$6.08 \cdot 10^{-1}$
ENST00000585673	ENSG00000172009	THOP1	7064	-0.55	$2.82 \cdot 10^{-1}$	$9.46 \cdot 10^{-3}$	$7.79 \cdot 10^{-1}$
ENST00000528164	ENSG00000151366	NDUFC2	4718	-0.54	$2.88 \cdot 10^{-1}$	$1.18 \cdot 10^{-2}$	$8.37 \cdot 10^{-1}$
ENST00000294507	ENSG00000162511	LAPTM5	7805	-0.54	$2.88 \cdot 10^{-1}$	$1.40 \cdot 10^{-3}$	$3.93 \cdot 10^{-1}$
ENST00000314340	ENSG00000177674	AGTRAP	57085	-0.54	$2.88 \cdot 10^{-1}$	$3.35 \cdot 10^{-2}$	1.00
ENST00000370406	ENSG00000197021	EOLA2	541578	-0.54	$2.88 \cdot 10^{-1}$	$3.60 \cdot 10^{-2}$	1.00
ENST00000007264	ENSG00000007376	RPUSD1	113000	-0.54	$2.88 \cdot 10^{-1}$	$4.33 \cdot 10^{-2}$	1.00
ENST00000390645	ENSG00000188042	ARL4C	10123	-0.53	$2.95 \cdot 10^{-1}$	$1.12 \cdot 10^{-2}$	$8.19 \cdot 10^{-1}$
ENST00000359832	ENSG00000241468	ATP5MF	9551	-0.53	$2.95 \cdot 10^{-1}$	$1.96 \cdot 10^{-3}$	$4.19 \cdot 10^{-1}$
ENST00000324093	ENSG00000004399	PLXND1	23129	-0.53	$2.95 \cdot 10^{-1}$	$3.24 \cdot 10^{-2}$	1.00
ENST00000078429	ENSG00000088256	GNA11	2767	-0.53	$2.95 \cdot 10^{-1}$	$4.55 \cdot 10^{-2}$	1.00
ENST00000295760	ENSG00000163520	FBLN2	2199	-0.52	$3.02 \cdot 10^{-1}$	$1.02 \cdot 10^{-2}$	$8.12 \cdot 10^{-1}$
ENST00000610533	ENSG00000106628	POLD2	5425	-0.52	$3.02 \cdot 10^{-1}$	$2.94 \cdot 10^{-3}$	$4.67 \cdot 10^{-1}$
ENST00000640799	ENSG00000143612	C1orf43	25912	-0.52	$3.02 \cdot 10^{-1}$	$2.95 \cdot 10^{-2}$	1.00
ENST00000359530	ENSG00000047249	ATP6V1H	51606	-0.52	$3.02 \cdot 10^{-1}$	$3.67 \cdot 10^{-2}$	1.00
ENST00000393562	ENSG00000160211	G6PD	2539	-0.51	$3.09 \cdot 10^{-1}$	$1.48 \cdot 10^{-3}$	$3.93 \cdot 10^{-1}$
ENST00000380181	ENSG00000057608	GDI2	2665	-0.51	$3.09 \cdot 10^{-1}$	$2.72 \cdot 10^{-2}$	1.00
ENST00000468280	ENSG00000105568	PPP2R1A	5518	-0.51	$3.09 \cdot 10^{-1}$	$3.30 \cdot 10^{-2}$	1.00

ENST00000469678	ENSG00000163931	TKT	7086	-0.51	$3.09{\cdot}10^{-1}$	$4.28{\cdot}10^{-2}$	1.00
ENST00000481070	ENSG00000156113	KCNMA1	3778	-0.51	$3.09{\cdot}10^{-1}$	$4.51{\cdot}10^{-3}$	$5.83{\cdot}10^{-1}$
ENST00000278840	ENSG00000134824	FADS2	9415	-0.51	$3.09{\cdot}10^{-1}$	$5.05{\cdot}10^{-3}$	$6.08{\cdot}10^{-1}$
ENST00000526072	ENSG00000110651	CD81	975	-0.5	$3.16{\cdot}10^{-1}$	$1.22{\cdot}10^{-2}$	$8.50{\cdot}10^{-1}$
ENST00000349048	ENSG00000141959	PFKL	5211	-0.5	$3.16{\cdot}10^{-1}$	$1.94{\cdot}10^{-2}$	$9.75{\cdot}10^{-1}$
ENST00000297350	ENSG00000164761	TNFRSF11B	4982	-0.5	$3.16{\cdot}10^{-1}$	$2.26{\cdot}10^{-3}$	$4.36{\cdot}10^{-1}$
ENST00000301488	ENSG00000167797	CDK2AP2	10263	-0.5	$3.16{\cdot}10^{-1}$	$4.61{\cdot}10^{-2}$	1.00
ENST00000514825	ENSG00000170606	HSPA4	3308	0.51	3.24	$1.25{\cdot}10^{-2}$	$8.50{\cdot}10^{-1}$
ENST00000536661	ENSG00000150991	UBC	7316	0.51	3.24	$1.58{\cdot}10^{-2}$	$9.38{\cdot}10^{-1}$
ENST00000394803	ENSG00000109332	UBE2D3	7323	0.51	3.24	$3.13{\cdot}10^{-2}$	1.00
ENST00000511473	ENSG00000204628	RACK1	10399	0.51	3.24	$4.68{\cdot}10^{-2}$	1.00
ENST00000518918	ENSG00000172172	MRPL13	28998	0.52	3.31	$4.89{\cdot}10^{-2}$	1.00
ENST00000530109	ENSG00000104529	EEF1D	1936	0.53	3.39	$1.85{\cdot}10^{-2}$	$9.75{\cdot}10^{-1}$
ENST00000312521	ENSG00000174738	NR1D2	9975	0.53	3.39	$3.30{\cdot}10^{-2}$	1.00
ENST00000644549	ENSG00000122406	RPL5	6125	0.53	3.39	$3.76{\cdot}10^{-3}$	$5.35{\cdot}10^{-1}$
ENST00000225696	ENSG00000108559	NUP88	4927	0.54	3.47	$2.04{\cdot}10^{-2}$	$9.75{\cdot}10^{-1}$
ENST00000424390	ENSG00000117450	PRDX1	5052	0.56	3.63	$1.28{\cdot}10^{-2}$	$8.56{\cdot}10^{-1}$
ENST00000649542	ENSG00000117139	KDM5B	10765	0.56	3.63	$2.33{\cdot}10^{-2}$	1.00
ENST00000267869	ENSG00000140307	GTF2A2	2958	0.57	3.72	$3.21{\cdot}10^{-2}$	1.00
ENST00000415325	ENSG00000091140	SLC25A23	1738	0.57	3.72	$3.33{\cdot}10^{-2}$	1.00
ENST00000535401	ENSG00000166741	NNMT	4837	0.58	3.80	$4.69{\cdot}10^{-2}$	1.00
ENST00000431973	ENSG00000109917	ZPR1	8882	0.58	3.80	$4.84{\cdot}10^{-2}$	1.00
ENST00000628419	ENSG00000124596	OARD1	221443	0.59	3.89	$3.24{\cdot}10^{-2}$	1.00
ENST00000546485	ENSG00000229117	RPL41	6171	0.59	3.89	$3.30{\cdot}10^{-2}$	1.00
ENST00000366528	ENSG00000203667	COX20	116228	0.6	3.98	$2.42{\cdot}10^{-2}$	1.00
ENST00000503445	ENSG00000153037	SRP19	6728	0.6	3.98	$2.69{\cdot}10^{-2}$	1.00
ENST00000578968	ENSG00000232956	SNHG15	285958	0.61	4.07	$1.08{\cdot}10^{-2}$	$8.12{\cdot}10^{-1}$
ENST00000433877	ENSG00000111011	RSRC2	65117	0.61	4.07	$3.72{\cdot}10^{-2}$	1.00
ENST00000392720	ENSG00000196704	AMZ2	51321	0.63	4.27	$1.07{\cdot}10^{-2}$	$8.12{\cdot}10^{-1}$
ENST00000395837	ENSG00000170315	UBB	7314	0.63	4.27	$1.91{\cdot}10^{-2}$	$9.75{\cdot}10^{-1}$
ENST00000521346	ENSG00000169139	UBE2V2	7336	0.64	4.37	$2.98{\cdot}10^{-2}$	1.00

ENST00000299964	ENSG00000166741	NNMT	4837	0.65	4.47	$4.09 \cdot 10^{-2}$	1.00
ENST00000420536	ENSG00000100316	RPL3	6122	0.65	4.47	$4.74 \cdot 10^{-2}$	1.00
ENST00000615790	ENSG00000242265	PEG10	23089	0.66	4.57	$2.06 \cdot 10^{-2}$	$9.75 \cdot 10^{-1}$
ENST00000479271	ENSG00000135829	DHX9	1660	0.66	4.57	$2.66 \cdot 10^{-2}$	1.00
ENST00000510628	ENSG00000163683	SMIM14	201895	0.67	4.68	$2.24 \cdot 10^{-2}$	$9.88 \cdot 10^{-1}$
ENST00000537646	ENSG00000215021	PHB2	11331	0.67	4.68	$6.54 \cdot 10^{-3}$	$6.59 \cdot 10^{-1}$
ENST00000525976	ENSG00000105397	TYK2	7297	0.68	4.79	$4.23 \cdot 10^{-2}$	1.00
ENST00000361556	ENSG00000117859	OSBPL9	114883	0.69	4.90	$1.35 \cdot 10^{-2}$	$8.71 \cdot 10^{-1}$
ENST00000560773	ENSG00000140319	SRP14	6727	0.69	4.90	$1.54 \cdot 10^{-2}$	$9.36 \cdot 10^{-1}$
ENST00000554963	ENSG00000133997	MED6	10001	0.69	4.90	$3.77 \cdot 10^{-2}$	1.00
ENST00000437329	ENSG00000118705	RPN2	6185	0.7	5.01	$3.35 \cdot 10^{-2}$	1.00
ENST00000436452	ENSG00000100129	EIF3L	51386	0.7	5.01	$3.66 \cdot 10^{-5}$	$5.10 \cdot 10^{-2}$
ENST00000394666	ENSG00000170634	ACYP2	98	0.7	5.01	$4.34 \cdot 10^{-2}$	1.00
ENST00000346473	ENSG00000175197	DDIT3	1649	0.71	5.13	$1.81 \cdot 10^{-2}$	$9.67 \cdot 10^{-1}$
ENST00000303698	ENSG00000169599	NFU1	27247	0.71	5.13	$3.18 \cdot 10^{-2}$	1.00
ENST00000545322	ENSG00000176731	RBIS	401466	0.72	5.25	$1.12 \cdot 10^{-2}$	$8.19 \cdot 10^{-1}$
ENST00000544052	ENSG00000092108	SCFD1	23256	0.72	5.25	$2.47 \cdot 10^{-2}$	1.00
ENST00000621269	ENSG00000066427	ATXN3	4287	0.72	5.25	$2.96 \cdot 10^{-2}$	1.00
ENST00000396774	ENSG00000047249	ATP6V1H	51606	0.72	5.25	$3.06 \cdot 10^{-2}$	1.00
ENST00000420643	ENSG00000119487	MAPKAP1	79109	0.73	5.37	$4.15 \cdot 10^{-2}$	1.00
ENST00000566751	ENSG00000141002	TCF25	22980	0.74	5.50	$2.07 \cdot 10^{-2}$	$9.75 \cdot 10^{-1}$
ENST00000357814	ENSG00000109065	NAT9	26151	0.74	5.50	$3.63 \cdot 10^{-2}$	1.00
ENST00000415213	ENSG00000140299	BNIP2	663	0.75	5.62	$1.18 \cdot 10^{-2}$	$8.37 \cdot 10^{-1}$
ENST00000356464	ENSG00000197728	RPS26	6231	0.76	5.75	$4.81 \cdot 10^{-2}$	1.00
ENST00000453195	ENSG00000234127	TRIM26	7726	0.77	5.89	$1.34 \cdot 10^{-2}$	$8.71 \cdot 10^{-1}$
ENST00000473611	ENSG00000227057	WDR46	9277	0.77	5.89	$3.57 \cdot 10^{-2}$	1.00
ENST00000398514	ENSG00000113657	DPYSL3	1809	0.78	6.03	$3.04 \cdot 10^{-2}$	1.00
ENST00000338145	ENSG00000109332	UBE2D3	7323	0.79	6.17	$5.94 \cdot 10^{-3}$	$6.41 \cdot 10^{-1}$
ENST00000474315	ENSG00000101365	IDH3B	3420	0.8	6.31	$8.69 \cdot 10^{-3}$	$7.54 \cdot 10^{-1}$
ENST00000650043	ENSG00000168216	LMBRD1	55788	0.81	6.46	$2.45 \cdot 10^{-2}$	1.00
ENST00000414108	ENSG00000095787	WAC	51322	0.82	6.61	$2.85 \cdot 10^{-2}$	1.00

ENST00000428648	ENSG00000154978	VOPP1	81552	0.82	6.61	$4.14\cdot10^{-2}$	1.00
ENST00000513807	ENSG00000164209	SLC25A46	91137	0.83	6.76	$1.53\cdot10^{-2}$	$9.35\cdot10^{-1}$
ENST00000368715	ENSG00000196154	S100A4	6275	0.87	7.41	$9.81\cdot10^{-3}$	$7.91\cdot10^{-1}$
ENST00000399683	ENSG00000101220	C20orf27	54976	0.88	7.59	$1.81\cdot10^{-3}$	$4.19\cdot10^{-1}$
ENST00000514914	ENSG00000205571	SMN2	6607	0.88	7.59	$3.19\cdot10^{-2}$	1.00
ENST00000530076	ENSG00000131238	PPT1	5538	0.89	7.76	$1.93\cdot10^{-2}$	$9.75\cdot10^{-1}$
ENST00000467560	ENSG00000171953	ATPAF2	91647	0.89	7.76	$8.88\cdot10^{-3}$	$7.54\cdot10^{-1}$
ENST00000439628	ENSG00000235109	ZSCAN31	64288	0.9	7.94	$1.13\cdot10^{-3}$	$3.59\cdot10^{-1}$
ENST00000530293	ENSG00000186174	BCL9L	283149	0.92	8.32	$2.20\cdot10^{-2}$	$9.88\cdot10^{-1}$
ENST00000463074	ENSG00000135930	EIF4E2	9470	-0.92	8.32	$3.35\cdot10^{-2}$	1.00
ENST00000549258	ENSG00000134294	SLC38A2	54407	0.92	8.32	$3.75\cdot10^{-4}$	$2.09\cdot10^{-1}$
ENST00000406109	ENSG00000106399	RPA3	6119	0.94	8.71	$1.52\cdot10^{-2}$	$9.33\cdot10^{-1}$
ENST00000380084	ENSG00000102054	RBBP7	5931	0.94	8.71	$4.11\cdot10^{-2}$	1.00
ENST00000643859	ENSG00000065883	CDK13	8621	0.94	8.71	$4.25\cdot10^{-2}$	1.00
ENST00000362076	ENSG00000143612	C1orf43	25912	0.94	8.71	$6.46\cdot10^{-3}$	$6.59\cdot10^{-1}$
ENST00000587560	ENSG00000132475	H3-3B	3021	0.94	8.71	$7.21\cdot10^{-3}$	$6.90\cdot10^{-1}$
ENST00000584008	ENSG00000197170	PSMD12	5718	0.96	9.12	$6.93\cdot10^{-3}$	$6.77\cdot10^{-1}$
ENST00000472348	ENSG00000106333	PCOLCE	5118	0.98	9.55	$2.15\cdot10^{-2}$	$9.79\cdot10^{-1}$
ENST00000481094	ENSG00000189159	JPT1	51155	0.98	9.55	$4.74\cdot10^{-2}$	1.00
ENST00000517486	ENSG00000197043	ANXA6	309	1.04	$1.10\cdot10^{1}$	$4.46\cdot10^{-2}$	1.00
ENST00000559674	ENSG00000140575	IQGAP1	8826	1.1	$1.26\cdot10^{1}$	$2.13\cdot10^{-3}$	$4.31\cdot10^{-1}$
ENST00000467767	ENSG00000173726	TOMM20	9804	1.1	$1.26\cdot10^{1}$	$3.56\cdot10^{-3}$	$5.24\cdot10^{-1}$
ENST00000345042	ENSG00000144381	HSPD1	3329	1.1	$1.26\cdot10^{1}$	$3.81\cdot10^{-3}$	$5.35\cdot10^{-1}$
ENST00000617482	ENSG00000182919	C11orf54	28970	1.1	$1.26\cdot10^{1}$	$7.54\cdot10^{-3}$	$7.05\cdot10^{-1}$
ENST00000556330	ENSG00000100650	SRSF5	6430	1.14	$1.38\cdot10^{1}$	$2.56\cdot10^{-3}$	$4.57\cdot10^{-1}$
ENST00000369472	ENSG00000146278	PNRC1	10957	1.15	$1.41\cdot10^{1}$	$1.53\cdot10^{-3}$	$3.93\cdot10^{-1}$
ENST00000532702	ENSG00000161016	RPL8	6132	1.2	$1.58\cdot10^{1}$	$2.45\cdot10^{-4}$	$1.61\cdot10^{-1}$
ENST00000467662	ENSG00000146112	PPP1R18	107987457	1.25	$1.78\cdot10^{1}$	$1.56\cdot10^{-2}$	$9.38\cdot10^{-1}$
ENST00000397053	ENSG00000151461	UPF2	26019	1.26	$1.82\cdot10^{1}$	$1.31\cdot10^{-3}$	$3.93\cdot10^{-1}$
ENST00000476344	ENSG00000178096	BOLA1	51027	1.29	$1.95\cdot10^{1}$	$1.48\cdot10^{-2}$	$9.33\cdot10^{-1}$
ENST00000463574	ENSG00000234745	HLA-B	3106	1.29	$1.95\cdot10^{1}$	$3.49\cdot10^{-3}$	$5.24\cdot10^{-1}$

ENST00000487799	ENSG00000144895	EIF2A	83939	1.3	$2.00\cdot10^{1}$	$2.19\cdot10^{-4}$	$1.61\cdot10^{-1}$
ENST00000482510	ENSG00000163636	PSMD6	9861	1.33	$2.14\cdot10^{1}$	$2.12\cdot10^{-2}$	$9.75\cdot10^{-1}$
ENST00000530188	ENSG00000175573	C11orf68	83638	1.35	$2.24\cdot10^{1}$	$2.46\cdot10^{-4}$	$1.61\cdot10^{-1}$
ENST00000463142	ENSG00000133606	MKRN1	23608	1.36	$2.29\cdot10^{1}$	$6.21\cdot10^{-4}$	$2.56\cdot10^{-1}$
ENST00000375633	ENSG00000204387	SNHG32	50854	1.39	$2.45\cdot10^{1}$	$3.59\cdot10^{-3}$	$5.24\cdot10^{-1}$
ENST00000564316	ENSG00000103266	STUB1	10273	1.46	$2.88\cdot10^{1}$	$2.31\cdot10^{-2}$	$9.99\cdot10^{-1}$
ENST00000433473	ENSG00000131238	PPT1	5538	1.59	$3.89\cdot10^{1}$	$2.02\cdot10^{-2}$	$9.75\cdot10^{-1}$
ENST00000555914	ENSG00000092199	HNRNPC	3183	1.61	$4.07\cdot10^{1}$	$1.93\cdot10^{-6}$	$5.44\cdot10^{-3}$
ENST00000583848	ENSG00000072778	ACADVL	37	1.63	$4.27\cdot10^{1}$	$1.05\cdot10^{-2}$	$8.12\cdot10^{-1}$
ENST00000508103	ENSG00000169045	HNRNPH1	3187	1.94	$8.71\cdot10^{1}$	$1.97\cdot10^{-8}$	$1.10\cdot10^{-4}$
ENST00000504687	ENSG00000160789	LMNA	4000	2.17	$1.48\cdot10^{2}$	$1.95\cdot10^{-6}$	$5.44\cdot10^{-3}$
ENST00000424454	ENSG00000095787	WAC	51322	2.25	$1.78\cdot10^{2}$	$5.26\cdot10^{-3}$	$6.10\cdot10^{-1}$
ENST00000577731	ENSG00000161960	EIF4A1	1973	2.41	$2.57\cdot10^{2}$	$9.00\cdot10^{-5}$	$1.00\cdot10^{-1}$
ENST00000579649	ENSG00000130255	RPL36	25873	3.6	$3.98\cdot10^{3}$	$4.14\cdot10^{-3}$	$5.50\cdot10^{-1}$
ENST00000364228	ENSG00000201098	RNY1	6084	10.5	$3.16\cdot10^{10}$	$1.42\cdot10^{-12}$	$1.58\cdot10^{-8}$
